# TIST: Transcriptome and Histopathological Image Integrative Analysis for Spatial Transcriptomics

**DOI:** 10.1016/j.gpb.2022.11.012

**Published:** 2022-12-19

**Authors:** Yiran Shan, Qian Zhang, Wenbo Guo, Yanhong Wu, Yuxin Miao, Hongyi Xin, Qiuyu Lian, Jin Gu

**Affiliations:** 1 MOE Key Laboratory of Bioinformatics, BNRIST Bioinformatics Division, Department of Automation, Tsinghua University, Beijing 100084, China; 2 UM-SJTU Joint Institute, Shanghai Jiao Tong University, Shanghai 200240, China; 3 Department of Automation, Shanghai Jiao Tong University, Shanghai 200240, China

**Keywords:** Spatial transcriptomics, Multimodal information integration, Network-based analysis, Spatial cluster identification, Gene expression enhancement

## Abstract

Sequencing-based **spatial transcriptomics** (ST) is an emerging technology to study *in situ* gene expression patterns at the whole-genome scale. Currently, ST data analysis is still complicated by high technical noises and low resolution. In addition to the transcriptomic data, matched histopathological images are usually generated for the same tissue sample along the ST experiment. The matched high-resolution histopathological images provide complementary cellular phenotypical information, providing an opportunity to mitigate the noises in ST data. We present a novel ST data analysis method called transcriptome and histopathological image integrative analysis for ST (TIST), which enables the identification of spatial clusters (SCs) and the enhancement of spatial gene expression patterns by integrative analysis of matched transcriptomic data and images. TIST devises a histopathological feature extraction method based on Markov random field (MRF) to learn the cellular features from histopathological images, and integrates them with the transcriptomic data and location information as a network, termed TIST-net. Based on TIST-net, SCs are identified by a random walk-based strategy, and gene expression patterns are enhanced by neighborhood smoothing. We benchmark TIST on both simulated datasets and 32 real samples against several state-of-the-art methods. Results show that TIST is robust to technical noises on multiple analysis tasks for sequencing-based ST data and can find interesting microstructures in different biological scenarios. TIST is available at http://lifeome.net/software/tist/ and https://ngdc.cncb.ac.cn/biocode/tools/BT007317.

## Introduction

Spatial information is crucial for understanding cellular functions in complex physiological and pathological processes. Recent advances in spatial transcriptomics (ST) technologies have made it feasible to achieve concurrent quantification and localization of messenger RNA (mRNA) molecules in tissues [1]. Widely used ST technologies can generally be classified into two categories: fluorescence *in situ* hybridization-based ST (FISH-ST) [2] and sequencing after *in situ* capture-based ST (SEQ-ST). The SEQ-ST techniques, such as the Visium Spatial Gene Expression, Slide-seq [3], Seq-Scope [4], and Stereo-seq [5], can achieve genome-scale transcriptomic profiling without known transcript information.

Despite the obvious advantages of supplementing mRNA sequencing with spatial information, current SEQ-ST techniques still suffer from several technical limitations, such as high dropout rate (DR) and severe molecular diffusion, *i.e.*, mRNA molecules from one spot diffuse to nearby spots during the permeabilization process. These technical noises heavily impact the downstream computational analyses. A few computational methods tried to integrate single-cell RNA sequencing (scRNA-seq) data to reduce the noises [6], [7]. However, the scRNA-seq data are frequently unavailable for the matched sample, which limits the applicability of these methods.

Incorporation of multi-modality information into the transcriptomic analysis is promising to mitigate the technical limitations of SEQ-ST. Existing approaches could be divided into two categories. The first category integrates the spatial locations and gene expression profiles based on diverse models and shows improvement in multiple analytical tasks. For example, BayesSpace [8] based on a Bayesian statistical method, STAGATE [9] based on graph attention auto-encoder, and STEEL [10] based on manifold learning jointly model expression profiles and the corresponding locations, improve the clustering performance of ST. SEDR [11] uses deep auto-encoder to co-embed gene expression and spatial location for low-dimensional representation. SPARK [12] adopts a generalized linear spatial model with a variety of spatial kernels to detect spatial expression patterns. The second category introduces the matched histopathological images generated by hematoxylin and eosin (H&E) staining into the model in addition to gene expression and spatial location. As the images are taken before the ST experiment, they capture the raw cell features without the molecular diffusion effect. Besides, the images have higher resolution than ST data and provide additional information on cellular phenotypes. For example, stLearn [13] leverages morphological similarities to normalize gene expression profiles before clustering and trajectory analysis. SpaGCN [14] integrates the three data modalities through graph convolution to identify spatial domains of coherent expression and histopathology. Both show potential to alleviate the impact of technical limitations.

However, the integrative analysis of ST data with histopathological images remains a challenging computational task. Although the transcriptomic data and histopathological images shared linked connections [15], they have different data formats, dimensions, resolutions, and noise levels. A unified method is needed to integratedly analyze the two data modalities for identifying and annotating the spatial clusters (SCs; *i.e.*, targeted tissue regions with distinct gene expression patterns, histopathological features, and biological functions), as well as for enhancing the gene expression patterns by imputing and correcting the raw ST data caused by under-sampling and molecular diffusion.

In this study, we propose an analytical tool suite for ST data named transcriptome and histopathological image integrative analysis for ST (TIST), which includes an SC identification module and a gene enhancement module. Previous work has employed an undirected probabilistic graph model for ST data analytics [8]; TIST expands this model by network-based data fusion, incorporating transcriptomic, histopathological, and adjacency information into a single spot-wise similarity network. From the fused similarity network, TIST identifies SCs through a customized random walk algorithm and enhances gene expression through a neighborhood smoothing algorithm. We benchmark TIST against several state-of-the-art ST analytical methods on both simulated datasets and real datasets. Results show that TIST can drastically reduce the impact of molecular diffusion and high mRNA DR, thus improving the performance of several downstream tasks, including SC identification, co-expression analyses, and spatially differentially expressed gene (SDEG) analyses.

## Method

### Overview of TIST

**Figure 1:**
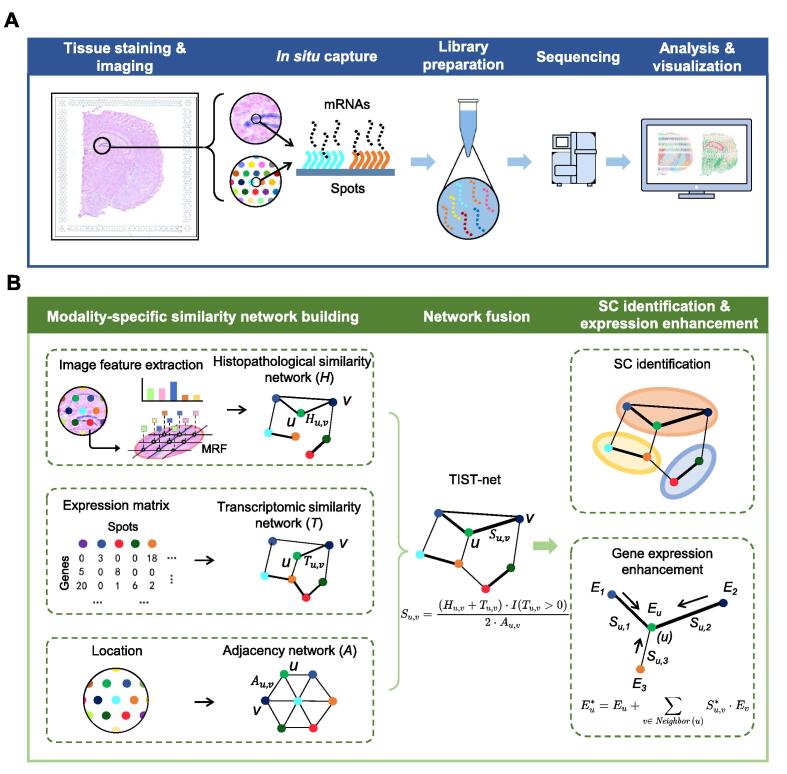
**Workflow of SEQ-ST techniques and TIST** **A.** The SEQ-ST workflow. The tissue section is first positioned on a glass slide printed with arrayed spatially barcoded RT primers. H&E staining and brightfield imaging are then conducted to obtain the histopathological image of the tissue section. In the permeabilization step, mRNAs diffuse and hybridize with local RT primers. After the *in situ* RT, cDNAs are synthesized and tagged with spatial barcodes. Finally, these spatially barcoded cDNAs are extracted and pooled for *ex situ* library preparation and sequencing. **B.** The key steps of TIST. TIST takes three modal information as inputs, including histopathological images, transcriptomic data, and spot locations, to construct three networks individually, followed by fusing them into a single network named TIST-net. Based on the fused network, TIST can identify SCs and enhance gene expression patterns. ST, spatial transcriptomics; H&E, hematoxylin and eosin; mRNA, messenger RNA; TIST, transcriptome and histopathological image integrative analysis for ST; SC, spatial cluster; SEQ-ST, *in situ* capture-based ST; RT, reverse transcription; MRF, Markov random field.

Current SEQ-ST techniques are generally powered by spatially barcoded probes to capture RNA transcripts *in situ* and label them with location information (Figure 1A). To overcome the two major noises, the high DR and the molecular diffusion that occurs during the permeabilization process, we propose a computational suite named TIST to improve the identification of SCs and the enhancement of gene expression by effectively integrating ST data with histopathological images (Figure 1B). By extracting features from each data modality, TIST separately builds three modality-specific networks: a histopathological similarity network generated from high-resolution H&E images by Markov random field (MRF) (Figure S1A and B), a transcriptomic similarity network generated from the gene expression data by shared nearest neighbor (SNN) [16], and a spatial adjacency network based on the spot locations. Then, TIST fuses the three networks into a multi-modality similarity network, termed TIST-net, for SC identification and gene expression enhancement. Finally, different simulation experiments are conducted to test the stability of TIST against two major noises, the diffusion noises during the permeabilization process, and the dropouts caused by low capture rate. Also, TIST is tested on a total of 32 real tissue slides (Table S1).

### Construction of the histopathological similarity network

To fully exploit the histopathological image, we build an effective image feature extraction method based on the MRF model and transform pixel-level data into spot-level representation. The H&E image is converted to grayscale, which has been demonstrated to be able to maintain morphological properties [17]. As shown in Figure 1B, MRF sets a hidden label for each pixel. The pixel labels are initialized by *K*-means and the total pixel cluster number K is a parameter (default value is set to 50). Considering third-order neighborhood, MRF maximizes the whole likelihood via Expectation-Maximization (EM) algorithm, grouping all pixels across the image into different clusters. We use the pixel label distribution over the region that an individual spot covers as the representation of spot-level histopathological features.

Then, we construct the image-based spot-wise similarity network. Let $w_{u}$ and $w_{v}$ denote the pixel label distribution of two spots *u* and *v*. The symmetrical Kullback–Leibler (KL) divergence *L_u,v_* between spots *u* and *v* is calculated as follows:

(1) $$\begin{array}{c} L_{u,v}=\frac{KL\left(w_{u}\|w_{v}\right)+KL\left(w_{v}\|w_{u}\right)}{2} \end{array}$$

(2) $$\begin{array}{c} KL\left(w_{u}\|w_{v}\right)=\sum_{i=1}^{K}w_{u,i}\log\frac{w_{v,i}}{w_{u,i}} \end{array}$$

(3) $$\begin{array}{c} KL\left(w_{v}\|w_{u}\right)=\sum_{i=1}^{K}w_{v,i}\log\frac{w_{u,i}}{w_{v,i}} \end{array}$$

Here, $w_{u,i}$ and $w_{v,i}$ denote the probability or proportion of pixels in spots *u* and *v* belonging to label *i*, respectively. The adjacency matrix of the histopathological similarity network, *H*, could be calculated as follows:

(4) $$\begin{array}{c} H_{u,v}=1-\min\left(1,L_{u,v}\right)=\left\{\begin{array}{c} 0,\;\mathrm{when}\;L_{u,v}>1\\ 1-L_{u,v},\;\mathrm{when}\;0<L_{u,v}<1 \end{array}\right) \end{array}$$

### Construction of the transcriptomic similarity network

We next construct a spot-wise transcriptomic similarity network. We first select the top 2000 highly variable genes using the FindVariableGenes function from the Seurat package in R and perform principal component analysis (PCA). With the top 50 principal components (PCs), we construct a SNN [16] graph using the FindNeighbors function from the Seurat package with the default settings. The edge weights of the SNN graph are used as the adjacency matrix of the transcriptomic similarity network *T*:

(5) $$\begin{array}{c} T_{u,v}=SNN_{weight\left(u,v\right)} \end{array}$$

### Construction of TIST-net

Finally, we integrate the histopathological similarity network *H* and the transcriptomic similarity network *T* together with the spatial locations of spots into a multi-modality network, termed TIST-net. The physical spatial distance between spots *u* and *v*, denoted as *A_u,v_*, is measured with Manhattan distance. The final adjacency matrix of TIST-net is defined as follows:

(6) $$\begin{array}{c} S_{u,v}=\frac{\left(H_{u,v}+T_{u,v}\right)\cdot I\left(T_{u,v}>0\right)}{2\cdot A_{u,v}\;} \end{array}$$

where *I* is the indicator function. *S_u,v_* is then normalized to 0–1 by:

(7) $$\begin{array}{c} S_{u,v}^{*}=\frac{S_{u,v}-\min\left(S\right)}{\max\left(S\right)-\min\left(S\right)} \end{array}$$

### Gene expression enhancement

Using the neighboring information in TIST-net, we could enhance gene expression by calculating the weighted average of the expression of immediate neighbors for a single spot. For any spot *u*, we define:

(8) $$\begin{array}{c} E_{u}^{*}=E_{u}+\underset{v\in Neighbor\left(u\right)}{\sum }S_{u,v}^{*}\cdot E_{v} \end{array}$$

Here, E* is the enhanced gene expression, and *E* is the raw gene expression. *Neighbor*(*u*) represents the immediate neighbors of spot *u* in TIST-net.

### Identification of SCs

TIST detects communities from TIST-net as SCs using Walktrap, a random walk-based algorithm [18]. Let *D* denotes the diagonal matrix of the node degrees and *S* denotes the edge weight matrix of TIST-net, so the transition probability matrix is calculated as *P* = *D***^−^**^1^*S*. The distance between spots *u* and *v*, denoted as *W_u,v_*, could be calculated as:

(9) $$\begin{array}{c} W_{u,v}=\left|\left|D^{-\frac{1}{2}}P_{u\cdot }^{t}-D^{-\frac{1}{2}}P_{v\cdot }^{t}\right|\right| \end{array}$$

where $P_{u\cdot }^{t}$ and $P_{v\cdot }^{t}$ are probability vectors of *t*-step random transition probability from spot *u* and spot *v* toward other spots, respectively, while *t* represents the step strength of random walk. *W_u,v_* is demonstrated to well quantify the structural similarities using random walk [18]. This distance, which captures the network community structure features, enables SC identification by hierarchical clustering.

### Simulation of diffusion effect

To mimic the diffusion effect of the permeabilization process, we use *D*(*u*|*u*_0_) to denote the diffusion effect produced by source spot *u*_0_, which acts on any other target spot *u*, based on Fick’s first law of diffusion [19]. We assume that each spot diffuses to other spots with lower concentrations and define:

(10) $$\begin{array}{c} C_{u_{0}}^{*}=C_{u_{0}}-\underset{i\neq 0}{\sum }D\left(u_{i}|u_{0}\right)+\underset{i\neq 0}{\sum }D\left(u_{0}|u_{i}\right) \end{array}$$

(11) $$\begin{array}{c} D\left(u|u_{0}\right)=J\left(u,u_{0}\right)\cdot k_{G}\left(u,u_{0}\right)\cdot C_{u_{0}} \end{array}$$

where $C_{u_{0}}$ represents original gene counts in spot *u*_0_; $C_{u_{0}}^{*}$ represents gene counts in spot *u*_0_ after diffusion; and *D*(*u*|*u*_0_) is the gene counts diffused from spot *u*_0_ to spot *u*. Based on Fick’s first law, *J* represents diffusion flux, defined as:

(12) $$\begin{array}{c} J\left(u,u_{0}\right)=\varphi \cdot \left(\left|\right|C_{u_{0}}{\left|\right|}_{1}\;-\;\left|\right|C_{u}{\left|\right|}_{1}\right) \end{array}$$

where *φ* represents the diffusion rate, which is related to temperature, liquid viscosity, and molecular size. We adjust *φ* in the range {0.1, 0.2, 0.3, …, 0.9} to obtain different simulation results. *k_G_*${\left(u,u_{0}\right)}^{⊤}$ is the Gaussian kernel function on the distance matrix, which can be written in the following form:

(13) $$\begin{array}{c} k_{G}\left(u,u_{0}\right)=\frac{1}{\sqrt{{\left(2\pi \right)}^{2}\left|\Sigma \right|}}exp\left(-\frac{1}{2}{\left(u-u_{0}\right)}^{⊤}\Sigma^{-1}\left(u-u_{0}\right)\right) \end{array}$$

(14) $$\begin{array}{c} \Sigma =\left(\begin{array}{cc} \sigma_{1}^{2} & \rho \sigma_{1}\sigma_{2}\\ \rho \sigma_{1}\sigma_{2} & \sigma_{2}^{2} \end{array}\right) \end{array}$$

where $u_{0}$ and u denote the spatial coordinates of the source spot *u*_0_ and target spot *u*, and |∑| is the determinant of the covariance matrix. We set *σ*_1_ = *σ*_2_ = 4 and *ρ* = 1 by default.

### Simulation of dropout

To simulate dropout events, we first randomly select elements in the gene expression matrix and set them to zero. The proportion of selected elements to all is defined as the DR, and we set this rate in the range {0.1, 0.2, 0.3, …, 0.9}. We then construct TIST-net. To fill in missing data, we use the average expression of adjacent non-zero vertices in TIST-net. Specifically, with the increase in the DR, the neighbor vertices of a missing vertex may also be lost. Therefore, we loop the fill operation above to ensure that all missed vertices are filled. Experiments show that the number of loops is positively related to the DR. We calculate KL divergence between two gene expression vectors before and after the dropout operation and define 1 − KL divergence as the dropout recovery score. A higher recovery score indicates better filling results.

### SDEG detection and evaluation

We detect SDEGs for each SC by TIST. Based on the good SC identification results of TIST, we predominantly detect those genes with spatial expression patterns that are displayed in certain SCs by means of the FindAllMarkers function in Seurat. We select the top 100 genes with the largest log_2_ fold change between the mean expression value in the target SC and others using the Wilcoxon rank-sum test to test differences. We utilize the significance level defined by SPARK to rank adjusted *P* values for SDEGs and select the top 100 genes for further analysis.

We reward genes with high total expression levels in adjacent spaces as more biologically meaningful and define *G_es_* to quantify the spatial enrichment effect as:

(15) $$\begin{array}{c} G_{es}=X\cdot N\cdot X^{T} \end{array}$$

Here, *X* represents the expression matrix with rows as genes and columns as spots, and *N* is the spot adjacent matrix of the k nearest neighbors (KNN) (selected according to the spatial location of the spot and set as 1 by default). For a specific gene *g*, its enrichment score equals to $\sum_{i}\sum_{j}\left(x_{g,i}\cdot x_{g,j}\cdot N_{ij}\right)$. This gene enrichment score is normalized to 0–1 as follows:

(16) $$\begin{array}{c} G_{es}^{*}=\frac{G_{es}-\min\left(G_{es}\right)}{\max\left(G_{es}\right)-\min\left(G_{es}\right)} \end{array}$$

### Quantitative indicators for evaluating clustering effects

To evaluate the similarity between the identified SC labels and the ground truth data labels, we use two commonly used quantitative metrics: Adjusted Rand Index (ARI) and Normalized Mutual Information (NMI):

(17) $$\begin{array}{c} \mathrm{ARI}=\frac{\sum_{\mathrm{ij}}\left(\frac{{SN\_C}_{\mathrm{ij}}}{2}\right)-\frac{\left[\sum_{i}\left(\frac{{SN\_P}_{i}}{2}\right)\sum_{j}\left(\frac{{SN\_T}_{j}}{2}\right)\right]}{\left(\frac{\mathrm{SN}}{2}\right)}}{\frac{1}{2}\left[\sum_{i}\left(\frac{{SN\_P}_{i}}{2}\right)+\sum_{j}\left(\frac{{SN\_T}_{j}}{2}\right)\right]-\frac{\left[\sum_{i}\left(\frac{{SN\_P}_{i}}{2}\right)\sum_{j}\left(\frac{{SN\_T}_{j}}{2}\right)\right]}{\left(\frac{\mathrm{SN}}{2}\right)}} \end{array}$$

(18) $$\begin{array}{c} \mathrm{NMI}=\frac{2\sum_{i}\sum_{j}\frac{SN\_C_{\mathrm{ij}}}{\mathrm{SN}}\cdot \log\left(\frac{\mathrm{SN}\cdot SN\_C_{\mathrm{ij}}}{SN\_P_{i}\cdot SN\_T_{j}}\right)}{-(\sum_{i}\frac{SN\_P_{i}}{\mathrm{SN}}\cdot \log\frac{SN\_P_{i}}{\mathrm{SN}}+\sum_{j}\frac{SN\_T_{j}}{\mathrm{SN}}\cdot \log\frac{SN\_T_{j}}{\mathrm{SN}})} \end{array}$$

Here, SN represents the number of all spots in one slice. ${SN\_P}_{i}$ represents the number of spots classified into the predicted cluster *i*, and ${SN\_T}_{j}$ represents the number of spots in the true cluster *j*. ${SN\_C}_{\mathrm{ij}}$ represents the number of overlapping spots between the predicted cluster *i* and the true cluster *j*. Both ARI and NMI reach the highest value at 1 and lowest score at 0, as the higher values indicate the better identification performance.

To quantitatively assess the effects of different clustering methods, we design both supervised and unsupervised indices. Regarding supervised classification accuracy *A_s_*, we define:

(19) $$\begin{array}{c} A_{s}=\frac{S_{\mathrm{consistent}}}{S_{\mathrm{all}}}\cdot 100\% \end{array}$$

Here, *S_consistent_* is the number of spots that belong to SCs identified consistent with those in the ground truth, and *S_all_* is the number of all spots in a dataset. For an annotated dataset, we first obtain labels of spots belonging to different SCs by different methods and then map those cluster labels to the ground truth by maximum matching and calculate the matching proportion.

The unsupervised modularity *Q* score is introduced to measure the clustering effects of networks constructed by different methods. The better clustering effects present more edges within the same clusters and fewer edges among different clusters. Here, we define:

(20) $$\begin{array}{c} Q=\sum_{c}\left(\frac{N_{\mathrm{inner}}}{N_{\mathrm{all}}}-{\left(\frac{2N_{\mathrm{inner}}+N_{\mathrm{outer}}}{2N_{\mathrm{all}}}\right)}^{2}\right) \end{array}$$

where *N_inner_* and *N_outer_* represent the numbers of edges whose vertices belong to the same clusters and different clusters, respectively. *N_all_* is the number of all edges in the network. *C* represents all clusters in the network. The value of *Q* is positively related to a better clustering effect. The range of the *Q* value is [−0.5,1). A previous study [20] has shown that a range [0.3,0.7] of *Q* represents the most satisfactory clustering effect.

### Data source

We benchmarked TIST on 32 real tissue slides by the Visium platform, including 11 10x Genomics officially released samples (https://www.10xgenomics.com/resources/datasets; Table S1) and 21 human primary liver cancer samples from our previous study [21]. Among these samples, one mouse cerebral cortex sample and one hepatocellular carcinoma (HCC) sample were used for the in-depth performance assessment.

### Data preprocessing

To extract the effective pixels of H&E staining images, we first converted them into grayscale and then fitted a mixed Gaussian model to identify the foreground pixels covered by tissue. For spatial RNA-seq data, we normalized the gene expression using the R package Seurat (v4.0.3) by the default method LogNormalize. Then, the expression of each gene was scaled to a standard normal distribution.

## Results

### TIST enables unsupervised identification of biologically meaningful tissue domains

Different regions in complex tissues or organs display distinct transcriptomic and histopathological patterns. We applied TIST to two datasets with well-annotated regions and confirmed that TIST could effectively incorporate histopathological information, leading to biologically meaningful SCs.

**Figure 2:**
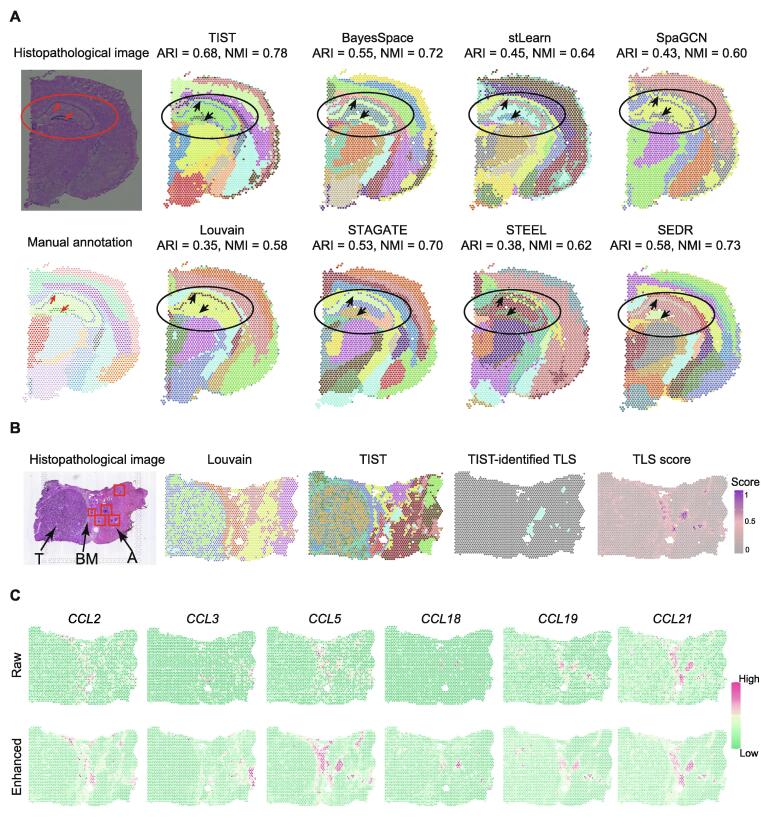
**SC identification results of TIST on mouse cerebral cortex dataset and HCC data** **A.** Comparisons among TIST and state-of-the-art methods on the mouse cerebral cortex dataset. The left column shows the histopathological image and manual annotation of this dataset. For details on the methods, see Table S3. SC identification results, as well as quantitative indexes ARI and NMI are shown, highlighting the hippocampus proper and the dentate gyrus in the hippocampus formation with red arrows. **B.** SC identification results on an HCC dataset. The first column shows the histopathological image of the HCC section, composed of T region on the left, BM region in the middle, and A liver region on the right. TLS regions with specific histopathological characteristics are marked with red boxes. The second and third columns show the SC identification results of Louvain and TIST, respectively. The fourth column displays one SC that TIST automatically identifies, which highly matches the TLS regions. The last column visualizes the TLS score of each spot based on the 12-chemokine signature scoring method. **C.** Raw expression patterns (upper panel) of typical 12-chemokine signature genes, including *CCL2*, *CCL3*, *CCL5*, *CCL18*, *CCL19*, and *CCL21*, and their TIST-enhanced expression patterns (bottom panel). ARI, adjusted Rand index; NMI, normalized mutual information; T, tumor; BM, basement membrane; A, adjacent; TLS, tertiary lymphatic structure; HCC, hepatocellular carcinoma.

We first analyzed the mouse cerebral cortex dataset shown in Figure 2A. We used manually annotated tissue domains guided by the reference atlas diagram from Allen Institute for Brain Science [22] as the ground truth (Figure 2A, Figure S2A; Table S2). The structure of the cerebral cortex is highly complex, such that adjacent areas within the cortex might be entirely different in both cell composition and function. For example, the dentate gyrus and the hippocampus proper (Ammon’s horn) are the two main and interlocking parts of the hippocampus formation [23]. The dentate gyrus, consisting of granule cells, contributes to the formation of new episodic memories and is one of the few structures where neurogenesis takes place in the adult brain [24]. The hippocampus proper plays a vital role in learning and memory. The principal cell layers of the two parts are indicated with arrows in the manual annotation results (Figure 2A).

Taking this manually annotated slide as a benchmarking sample, we compared TIST with several classical and recently published methods (Louvain [6], BayesSpace [8], STAGATE [9], STEEL [10], SEDR [11], stLearn [13], and SpaGCN [14]; and the feature comparisons among these methods are listed in Table S3) in terms of visualization results and two quantitative indexes, ARI and NMI. Of note, considering the sensitivity of Louvain to the resolution parameter, we tested Louvain in different resolutions and selected the parameter resolution at 0.3 in the comparison (Figure S2B–D). Parameters in other methods were set as default. Overall, the SCs identified by TIST highly agreed with the ground truth and TIST performed the best clustering accuracy with ARI = 0.68 and NMI = 0.78 (Figure 2A). Compared to Louvain, the classical clustering method developed for scRNA-seq data, all the other methods showed better performances. Beyond the two indexes, TIST can also better use the image features to identify relatively tiny tissue structures. For example, TIST accurately identified the predominant cell layers of the hippocampus and the dentate gyrus, compared to which the other methods did not precisely recover these important structures.

To assess the effects of each individual network in TIST, we re-calculated ARI and NMI by removing one network each time (the transcriptomic similarity network, histopathological similarity network, and physical spatial adjacency network, respectively) (Figure S3). We found that the hippocampus and the dentate gyrus cannot be identified accurately with any of the other two networks. Quantitatively, the performances were dropped by ∼ 14% in ARI and ∼ 6% in NMI when any one network was removed. These results suggest that the three networks contain complementary information and TIST successfully integrates these features to improve the accuracy for identifying relatively small tissue structures.

We further demonstrated the superior performance of TIST with an HCC dataset (HCC-1L in Table S1) [21]. The section covers the tumor border, enabling the dissection of immune infiltration. With histopathological knowledge, we separated the whole section into the tumor region on the left side, the basement membrane in the middle, and an adjacent liver region to the right of the section (Figure 2B). Notably, there are several tertiary lymphatic structure (TLS) regions in the adjacent liver region, marked with red boxes. TLS consists of ectopic aggregates of lymphoid cells in inflamed, infected, or tumoral tissues [25] and is reported to be closely related to tumor progression and metastasis, serving as a potential predictive marker for immunotherapy and prognosis [26]. Accurate identification of TLS regions from ST data is crucial for comprehensively depicting the tumor immune microenvironment. We annotated TLS regions with a 12-chemokine signature scoring method [27] as a reference and compared the TLS identification performance of TIST with that of Louvain. As shown in Figure 2B, TIST manages to identify a cluster of spatial domains denoted in seafoam color, which are consistent with annotated high TLS score domains based on 12-chemokine signature genes. However, the partition given by Louvain has difficulty matching the TLS annotations.

Moreover, we enhanced gene expression patterns through neighborhood smoothing based on TIST-net to interrogate whether the enhanced gene expression could mitigate the technical noises and better recapture the details of the biological tissue. Figure 2C shows six genes [27] with reported relationships to TLS and compares their raw expression (upper panel) and enhanced expression (bottom panel). Clearly, the enhanced expression patterns show much more obvious spatial patterns and match better with the TLS annotations in Figure 2B, confirming the capacity of TIST in integrating ST data with the histopathological image.

### TIST resists the noise induced by mRNA molecular diffusion

**Figure 3:**
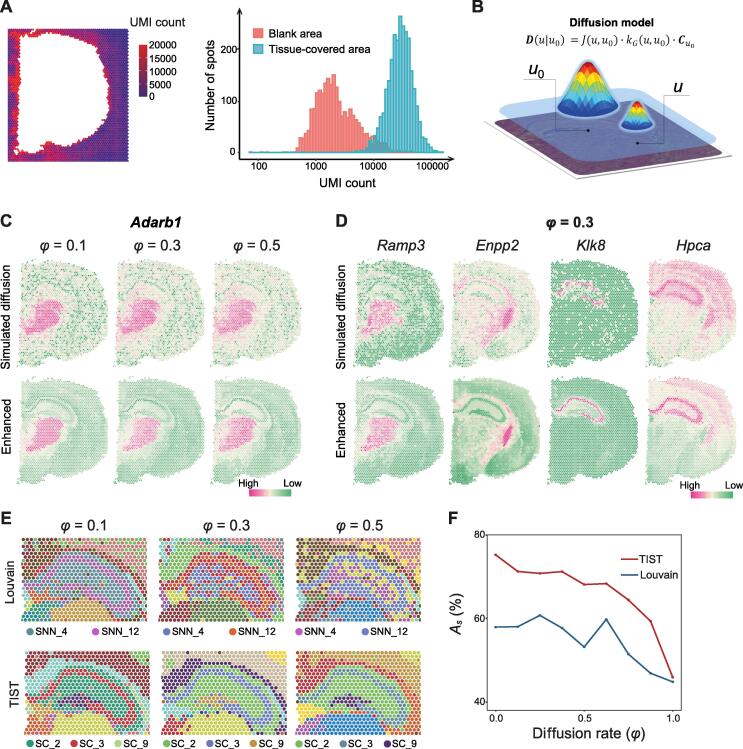
**Resistance of TIST to diffusion noise** **A.** Example of mRNA diffusion in SEQ-ST data. The left heatmap displays the UMI counts detected on the slide area not covered by tissue, induced by mRNA diffusion from the tissue-covered area. The right histogram shows the distributions of UMI counts detected in the blank area and in the tissue-covered area on the slide. **B.** Schematic of the simulation model of diffusion noise, describing the diffusion effect that is produced by the source spot *u*_0_ and acts on any other target spot *u*. The peak height represents the concentrations of mRNA molecules of a certain gene at the matching spot. **C.** Simulated expression patterns of the marker gene *Adarb1* with different settings of diffusion rate (upper panel) and the corresponding TIST-enhanced expression patterns (lower panel). **D.** Simulated expression patterns of the marker genes *Ramp3*, *Enpp2*, *Klk8*, and *Hpca*, with a diffusion rate fixed at 0.3 (upper panel) and the corresponding TIST-enhanced expression patterns (lower panel). **E.** Performances of Louvain (upper panel) and TIST (lower panel) in identifying and segregating the hippocampus proper and the dentate gyrus at different simulated diffusion rates. **F.** SC identification accuracies of Louvain (blue line) and TIST (red line) on the whole dataset given different simulation diffusion rates. *φ*, diffusion rate; *A_s_*, identification accuracy; UMI, unique molecular identifier.

The diffusion of mRNA molecules during the permeabilization process brings in inevitable noises challenging ST data analysis but has not received enough attention. In the permeabilization step, mRNA molecules can diffuse from their original locations into adjacent spots, leading to inaccurate spatial expression patterns. To showcase the severity of mRNA diffusion, we inspected the blank area in the section without tissue coverage, where no unique molecular identifiers (UMIs) should be present. We observed 1.67%–23.6% (average 8.30%) UMIs detected in blank areas in all datasets (Table S1) and visualized the UMI counts in the blank area of the mouse cerebral cortex dataset in Figure 3A.

We developed a diffusion simulation method according to Fick’s first law of diffusion [19] and benchmarked TIST on simulated datasets with ground truth. Figure 3B shows that the simulated mRNA diffusion effect from the source spot *u*_0_ to any other target spot *u*, denoted *D*(*u*|*u*_0_), is negatively related to the distance between the two spots and positively related to the concentration gradient and diffusion rate *φ*. Here, the diffusion rate *φ* models the influence of potential diffusion-related factors, including temperature, liquid viscosity, and molecular size.

We showed resistance of TIST to diffusion noise with marker genes that are specifically expressed in certain tissue domains. *Adarb1*, reported to be associated with the nervous system [28], was found to be specifically expressed in the sensory-motor cortex-related zone of the thalamus (BS-IB-IORsm). We tested simulated *φ* ranging from 0.1 to 0.9 and exhibited the results in Figure 3C and Figure S4A. TIST-enhanced expression of *Adarb1* restored its spatial pattern even when the diffusion effect was tremendous (Figure 3C). We further investigated various genes marking different SCs with fixed diffusion rates (Figure 3D, Figure S4B). To further validate the fidelity of gene enhancement, we compared all these gene expression patterns with the well-annotated “reference” data from Allen Mouse Brain Atlas [22]. The enhanced expression patterns by TIST are highly consistent with the *in situ* hybridization (ISH) as well as immunofluorescence (IF) results in the atlas (Figure S5). Comparing TIST-enhanced profiles with raw expression patterns not undergoing any simulation diffusion, we could conclude that TIST-enhanced ST data well reserve the spatial expression patterns and enable stable recapitulation of tissue structures even with severe diffusion noise.

We further benchmarked TIST for SC identification and classification with simulated datasets at different diffusion rates and compared TIST with Louvain [6], a classical clustering method developed for scRNA-seq. The performance of TIST compared with that of Louvain in identifying the hippocampus proper and the dentate gyrus is shown in Figure 3E and Figure S5B. To fully evaluate SC identification performance of resisting diffusion noise, we investigated whether TIST could identify SCs from simulated diffusion datasets that well resemble the manually annotated results shown in Figure 2A. As shown in Figure 3F, TIST indeed achieves superior SC identification and classification performance compared to Louvain with higher recognition accuracy *A_s_* across all diffusion rate settings. An unsupervised modularity index was also adopted as the metric [20] and agreed that TIST performs better than Louvain at SC identification with diffusion noise (Figure S5C).

### TIST accommodates high DR

As with scRNA-seq, ST methods also suffer from a low mRNA capture rate, resulting in a large proportion of false zeros for expressed genes, which are called “dropout” events. Currently, SEQ-ST datasets can reach a median of 600 to 6000 genes detected per spot (Table S1). A high ratio of dropout would compromise the accuracy of downstream analyses. There is an urgent need to develop ST analysis methods to overcome high DR.

**Figure 4:**
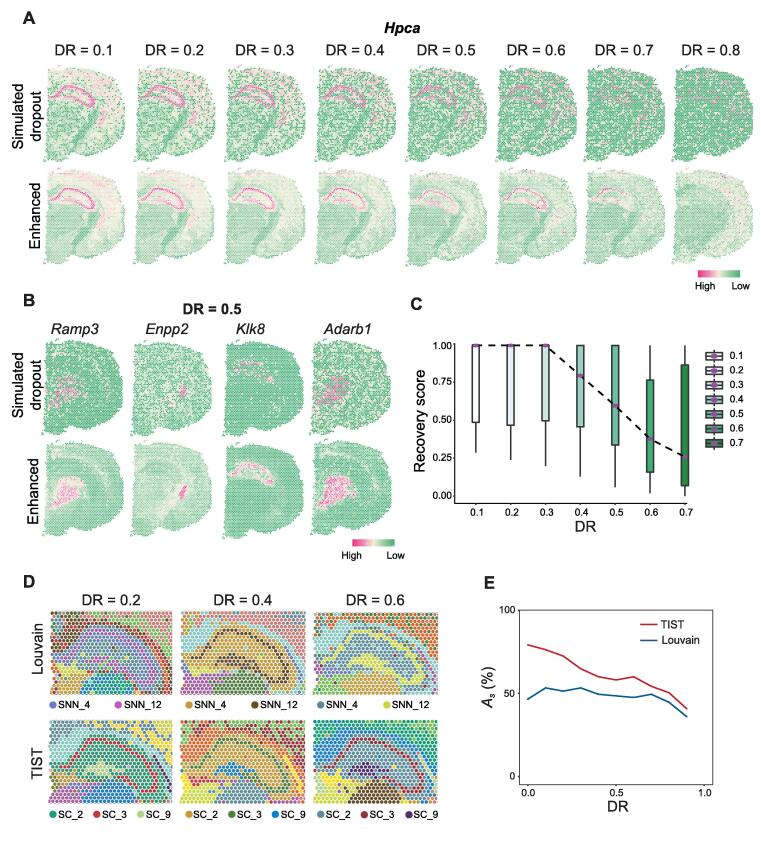
**Resistance of TIST to dropout noise** **A.** Simulated expression patterns of the marker gene *Hpca* with DRs ranging from 0.1 to 0.8 (upper panel) and the corresponding TIST-enhanced expression patterns (lower panel). **B.** Simulated expression patterns of the marker genes *Ramp3*, *Enpp2*, *Klk8*, and *Adrab1* at a fixed DR of 0.5 (upper panel) and the corresponding TIST-enhanced expression patterns (lower panel). **C.** Boxplot of recovery scores that TIST achieves on 49 genes reported to be related to brain activities under different simulated DRs. The dotted line connects the median recovery scores in each condition. **D.** Performances of Louvain (upper panel) and TIST (lower panel) in identifying and segregating the hippocampus proper and the dentate gyrus at different simulated DRs. **E.** SC identification accuracies of Louvain (blue line) and TIST (red line) on the whole dataset given different simulated DRs. DR, dropout rate.

First, *Hpca*, a specific marker gene of the hippocampus proper [29], was selected to test whether TIST could enhance gene expression effectively from ST data with different dropouts. We randomly removed 10%–90% of non-zero data in the original expression matrix and used TIST-net to fill and restore the expression patterns. As the DR increased, the aggregation effect of *Hpca* expression gradually weakened (Figure 4A). Its spatial expression pattern became increasingly difficult to discern based on raw data. TIST-enhanced expression maintained distinct domain enrichment until the DR reached 0.8 (Figure 4A, Figure S5A). Fixing the simulated DR as 0.5, we further inspected various marker genes; and the results are shown in Figure 4B and Figure S6A. In addition, we designed a recovery score to quantitively measure how well TIST-enhanced expression profiles from simulated dropout datasets resemble expression patterns before simulating dropouts. We selected 49 genes (Table S4) reported to be related to brain activities [22] for evaluation. As shown in Figure 4C, it would be difficult to recover gene expression with high fidelity if we dropped more than 40% of the expressed genes from the currently measured ST data.

Next, we evaluated the influence of dropout on SC identification. Using the example of the hippocampus proper and the dentate gyrus, we compared the performance of TIST against that of Louvain with simulated DRs ranging from 0.1 to 0.8. As shown in Figure 4D, Louvain always fails to distinguish the two regions, while TIST allows accurate identification and segregation of the two regions when the simulated DR is less than 0.6. We also quantitatively measured the SC identification performance on the whole mouse cerebral cortex dataset with both the supervised and unsupervised metrics (Figure 4E, Figure S6B), finding that the dropout resistance of TIST regarding SC identification significantly surpasses that of Louvain.

### TIST refines the detection of spatial expression patterns

The detection of SDEGs is a powerful way to link spatial regions with biological functions. Previous work has shown that SPARK, built upon a generalized linear spatial model with a variety of spatial kernels, is a powerful tool for revealing spatial expression patterns [12]. We thus compared TIST with SPARK in identifying SDEGs on the mouse cerebral cortex dataset.

**Figure 5:**
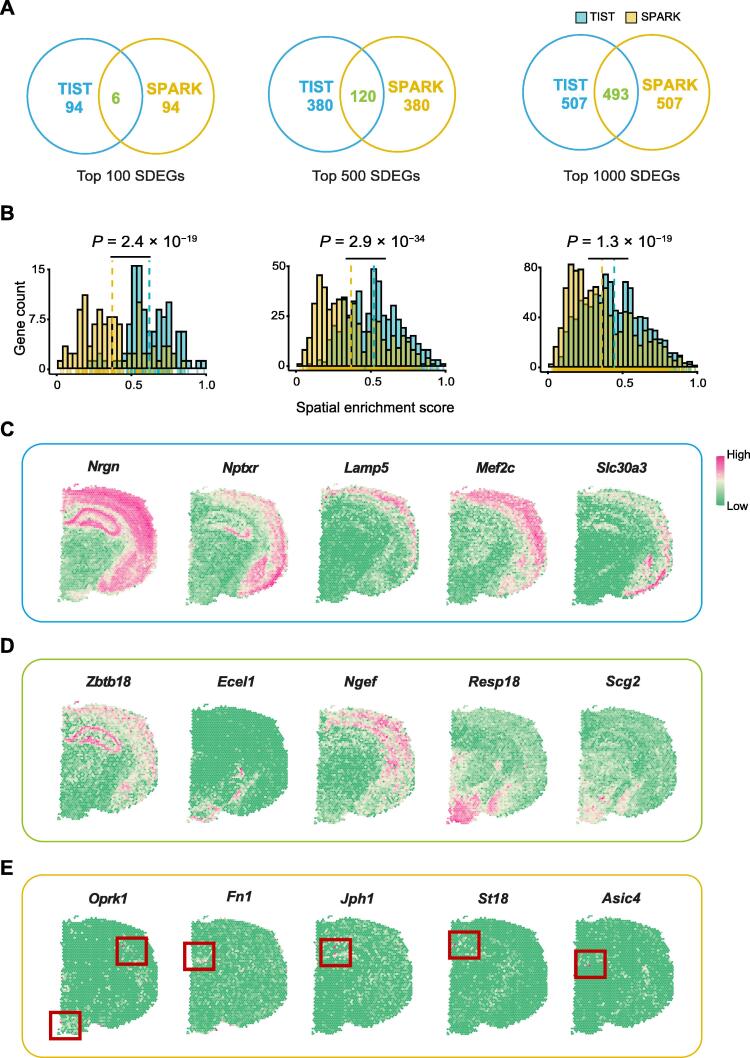
**Performance of TIST in facilitating SDEG detection** **A.** Venn diagram of the top 100, 500, and 1000 SDEGs identified by TIST and SPARK. **B.** Quantitative assessment of the top 100, 500, and 1000 SDEGs detected by TIST and SPARK. The yellow and blue dotted lines indicate average spatial enrichment scores of SPARK and TIST, respectively. *P* value is calculated by *t*-test. **C.** Spatial expression patterns of typical genes detected by TIST, but not by SPARK, in the top 100 SDEGs. **D.** Spatial expression patterns of typical genes commonly identified by both TIST and SPARK in the top 100 SDEGs. **E.** Spatial expression patterns of typical genes detected by SPARK, but not by TIST, in the top 100 SDEGs. Scattered spots with singularly high expression are marked with red boxes. SDEG, spatially differentially expressed gene.

We evaluated SDEG identification by comparing the top 100, top 500, and top 1000 SDEGs identified by TIST and SPARK. As shown in Figure 5A, only 6 genes were shared in the top 100 SDEGs, while the overlap increased as the total number increased, up to nearly 50% in the top 1000 SDEGs. We then used a spatial enrichment score to quantitatively evaluate the identified SDEGs. From the top 100 to top 1000, the SDEGs identified by TIST have significantly higher scores than SPARK (Figure 5B). To examine the identified genes with more detail, we visualized the expression patterns of the top 100 SDEGs by the two methods. Most SDEGs only identified by TIST showed strong spatial patterns (Figure 5C, Figure S7) as the 6 SDEGs identified by both methods (Figure 5D). However, many SDEGs only identified by SPARK seemed to be affected by data noises (Figure 5E, Figure S8). We further investigated the functional enrichments of the 94 exclusive SDEGs identified by TIST and SPARK, separately. Gene set analysis showed that the 94 SDEGs identified by TIST were significantly enriched in GO terms related to brain activities, but the 94 SDEGs of SPARK were enriched in non-related terms (Figure S9).

To further assess the reliability of gene enhancement, we additionally compared TIST with stLearn. Taking the mouse cerebral cortex sample as an example, we used the 49 marker genes related to brain activities for evaluation [22] and simulated dropout events on this dataset setting DR from 0.1 to 0.7, followed by applying TIST and stLearn to it. Comparing the two, we observed that TIST reaches greater median recovery scores than stLearn when DR is less than or equal to 0.3, whereas the scores of stLearn generally have smaller variance among the 49 genes than TIST (Figure S10A). Setting DR as 0.5, we then divided these genes into two groups: genes on which stLearn achieves greater recovery scores than TIST (stLearn > TIST) and the other genes on which TIST achieves better performance than stLearn (TIST > stLearn). We found that TIST performed better on genes with higher expression levels, lower DRs, and stronger spatial enrichment patterns (Figure S10B). It suggests that TIST has higher precision and stLearn has higher sensitivity. As summary, TIST could resist technical noises and identify more biologically meaningful spatial expression patterns.

### TIST benefits gene co-expression inference from ST data

It remains a great challenge to effectively incorporate spatial information from ST data and enable reliable detection of gene co-expression patterns in specific tissue regions. We show that TIST-identified SCs could facilitate the detection of region-specific gene co-expression patterns and shed light on complicated cell–cell communication in tissue.

**Figure 6:**
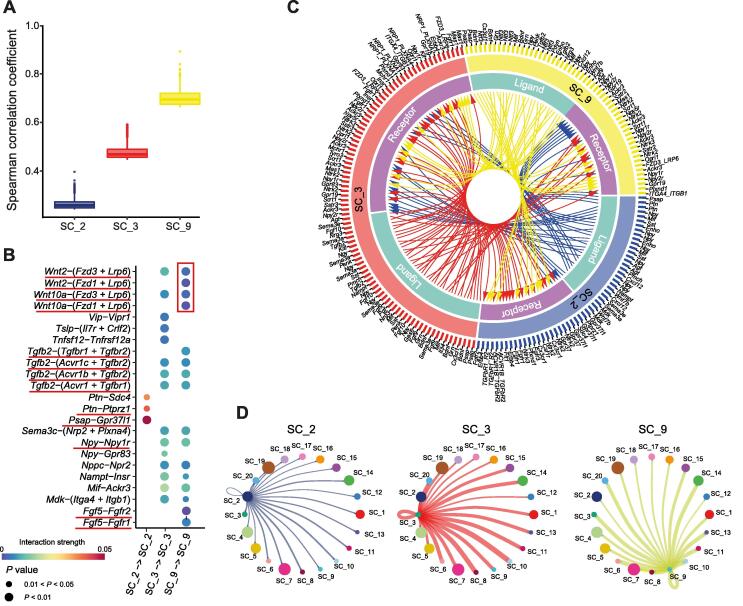
**Spatial co-expression analysis based on TIST** **A.** Bar plot showing the co-expression strength represented by Spearman correlation coefficients within three SCs (SC_2, SC_3, and SC_9) composing the hippocampus formation. **B.** Bubble plot of ligand–receptor pairs enriched within each of the three SCs. The area of the bubble represents the *P* value, while the color shows the interaction strength. We underline the ligand–receptor pairs supported by literature evidence. **C.** Circos plot showing ligand–receptor pairs among SC_2, SC_3, and SC_9. Curved arrows indicate the ligand–receptor relationships. Genes detected to be specific in different SCs are listed outside the circle. **D.** Communication strength between the three SCs and the others. The thickness of the curve represents the communication strength between two SCs that the curve connects. Spot size represents the number of enriched ligand–receptor pairs.

The hippocampus formation in the mouse cerebral cortex dataset is split by TIST into three SCs, including principal cell layers in the hippocampus proper (SC_3), the dentate gyrus (SC_9), and the remaining part (SC_2). We first compared the gene co-expression strength within the three SCs. Figure 6A visualizes the Spearman correlation coefficients between any pair of genes in the top 2000 SDEGs found by TIST. Gene co-expression is significantly stronger in the dentate gyrus region (SC_9) than in the other two regions, suggesting that the dentate gyrus undergoes highly precise regulation of gene expression, which might account for its critical role in neurogenesis. We further investigated cell communication with CellChat [30] to infer the strength of cell communication within and among the three SCs. Figure 6B shows significant ligand–receptor pairs within each SC, among which we marked cases supported by literature evidence with red underlines (Table S5). For instance, *Wnt* ligands binding to the combination of *Fzd1/3* and *Lrp6* co-receptors are reported to activate intracellular signaling and facilitate brain development as well as adult hippocampal neurogenesis [31], in line with the fact that the dentate gyrus region (SC_9), where neurogenesis takes place, enriches up to four related ligand–receptor pairs (marked with red box). Figure 6C visualizes significant ligand–receptor binding patterns among the three SCs. Specifically, the hippocampus proper (SC_3) communicates quite actively with the other two SCs, as the red color representing SC_3 takes up the highest proportion of ligand–receptor pairs in the outer ring. We further analyzed the communication between the three SCs and the other TIST SCs identified in the mouse cerebral cortex dataset. SC_3 maintained generally strong interactions with most SCs (Figure 6D). These observations echo the fact that the hippocampus properly contains hub neurons possessing widespread axonal arborizations [32].

### TIST adapts to diverse tissue types

**Figure 7:**
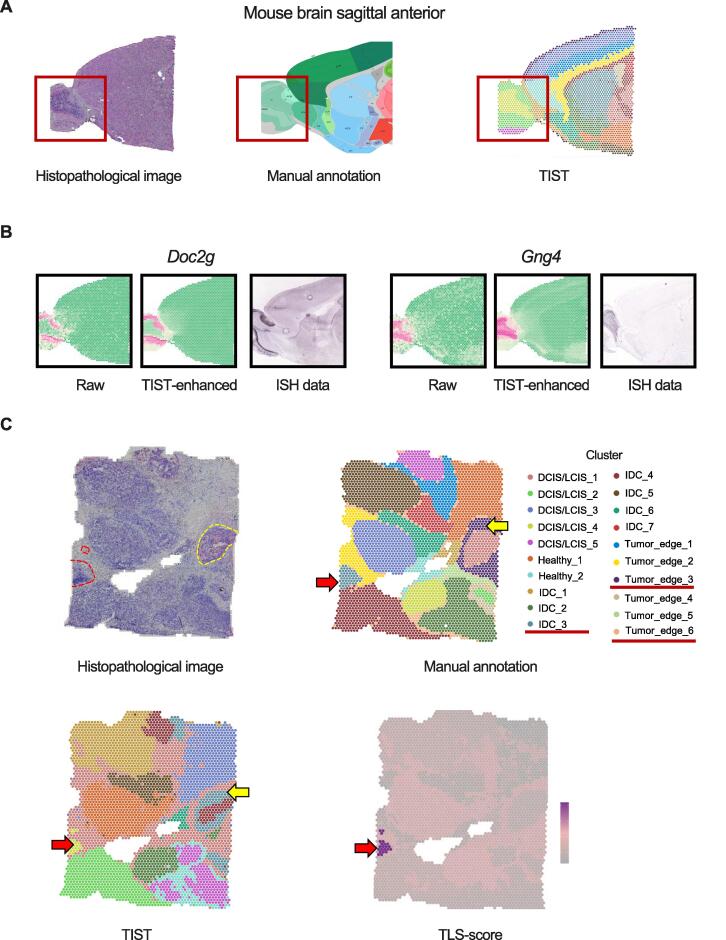
**SC identification results of TIST on datasets with diverse sample types** **A.** SC identification results of TIST on a mouse brain sagittal anterior dataset from 10x Genomics. Histopathological image, manual annotation, and SC identification of TIST are shown from left to right. The tiny structure OLF is emphasized in the red box. **B.** Gene enhancement results of TIST of typical genes *Doc2g* and *Gng4.* Raw expression pattern, TIST-enhanced expression pattern, and reference ISH image are listed in order. **C.** SC identification results of TIST on a human breast cancer dataset from 10x Genomics. The upper panels show the histopathological image and manual annotation, respectively. Two TLS regions were circled by red dashed lines and a refined tumor region was circled by a yellow dashed line. The SCs identified by TIST are shown at the left bottom. The TLS scores are visualized at the right bottom. OLF, olfactory area; ISH, *in situ* hybridization.

In addition to the mouse cerebral cortex and the HCC samples, we tested TIST with 32 samples in total (Table S1), covering diverse tissue types and different pathological conditions, including 11 10x Genomics officially released samples (Figure 7, Figure S11) and 21 human primary liver cancer samples from our previous study [21] (Figure S12). For the mouse brain sagittal anterior slide, TIST successfully detected the local structure olfactory area (OLF) in this tissue [22], and the enhanced gene expression patterns are consistent with the ISH data (Figure 7A and B). For another example of a human breast cancer slice, TIST found a small-size TLS next to a bigger one, IDC_3 region (circled by red dashed lines in Figure 7C), which was verified by a TLS score. Also, TIST refined the boundary between the Tumor_edge_3 and Tumor_edge_6 regions (circled by a yellow dashed line in Figure 7C). These results suggest that TIST is applicable to different tissue types.

## Discussion

We proposed TIST tailored toward SEQ-ST data by effectively integrating ST data and histopathological images via a network-based multimodal data fusion strategy. TIST unsupervisedly groups spots into SCs with coherent gene expression patterns, histopathological features, and biological functions, and enhances spatial expression signals from noisy ST data. The ablation experiments show that the three different data modalities provide complementary information, which has been successfully integrated by TIST. The features from histopathological images are helpful for identifying small structures by providing strong boundary penalties.

We reported that mRNA diffusion during the process of tissue permeabilization inevitably induces noise in ST data and blurs spatial expression patterns, which has not yet received enough attention. We demonstrated the severity of the diffusion noise and its interference in downstream ST analyses and showed that TIST could mitigate the technical noise by effectively integrating ST data and the matching histopathological images.

The rapid development of ST techniques has relieved some technical drawbacks, such as limited resolution, but many challenges remain to be solved [33]. Ideally, we want to obtain the expression profiles of individual cells together with their locations. For SEQ-ST, each spot captures mRNAs from proximal cells in the tissue section covering the slide. It would be difficult to obtain the mapping relationship between spots and cells and accurately recover expression profiles at the single-cell level from spot-level ST data. As histopathological images are taken prior to ST sequencing, they are expected to capture the ground truth cell arrangement and help obtain ideal single-cell ST data. However, reliable SC annotation and standardized positional mapping between ST data and image are still in need among popular SEQ-ST techniques. By now only 10x Genomics could provide them at the same time, so we are also working to add an automatic alignment method to mapping ST data with histopathological images for other platforms such as Seq-Scope [4] and Stereo-seq [5]. Collectively, it may be possible to extend TIST to realize accurate single-cell ST analysis given that TIST provides an excellent multimodal feature extraction and data fusion method.

## Code availability

The source code of TIST can be accessed at https://ngdc.cncb.ac.cn/biocode/tools/BT007317 and http://lifeome.net/software/tist/.

## CRediT author statement

**Yiran Shan:** Conceptualization, Methodology, Software, Validation, Formal analysis, Writing - original draft. **Qian Zhang:** Conceptualization, Investigation, Formal analysis, Writing - original draft. **Wenbo Guo:** Validation, Data curation. **Yanhong Wu:** Validation, Data curation. **Yuxin Miao:** Validation, Data curation. **Hongyi Xin:** Methodology, Supervision, Data curation. **Qiuyu Lian:** Conceptualization, Methodology, Supervision, Data curation, Writing - review & editing. **Jin Gu:** Conceptualization, Methodology, Investigation, Supervision, Writing - original draft, Writing - review & editing. All authors have read and approved the final manuscript.

## Competing interests

The authors have declared no competing interests.

## Supplementary material

Supplementary Figure S1 **Processing flow of TIST A.** Typical TIST processing on histopathology image. For the original image, we first obtain the outer boundary outline and explore the whole image to obtain inner lines. Boundary and edge information is then obtained. We use MRF to label this image and implement TIST to further recognize SCs. **B.** TIST process on the histopathology image of human colorectal cancer (upper left), mouse kidney (upper right), mouse brain sagittal anterior (lower left), mouse brain sagittal posterior (lower right) datasets. MRF, Markov random field.

Supplementary Figure S2 **Comparison of Louvain and TIST on the mouse cerebral cortex data A.** Histopathological image from 10X Visium platform and detailed manual annotation for predominant structures of mouse cerebral cortex from Allen Mouse Brain Atlas. **B.** SC identification results of Louvain at the best performance resolution 0.3 displayed over the spatial locations (left) and UMAP-transformed 2D pane (right). **C.** SC identification results of TIST displayed over the spatial locations (left) and the UMAP-transformed 2D pane (right). **D.** SC identification of Louvain with resolution adjusted in the range 0.1 to 2.0. The number of SCs is shown in the figure, while the clustering effect quantified by ARI is also shown. UMAP, uniform manifold approximation and projection; 2D, 2 dimensional.

Supplementary Figure S3 **Ablation experiment of TIST** Each component of TIST-net (three networks of histopathological information, transcriptomic information, and physical information) is removed once time, and TIST is then utilized on the mouse cerebral cortex dataset. Identification accuracy metrics of ARI and NMI are listed.

Supplementary Figure S4 **Supplementary results of diffusion simulation of TIST A.** Test of TIST gene enhancement for *Adarb1* when diffusion rate *φ* is from 0.1 to 0.9 excluding exhibited in Figure 3C, respectively. **B.** Spatial expression patterns of the specific marker genes *Ramp3, Enpp2, Klk8, Hpca, Cabp7,* and *Hap1* when setting the diffusion rate *φ* as 0.5.

Supplementary Figure S5 **Expression patterns of typical marker genes and comparison between TIST and Louvain in diffusion simulation A.** Raw expression, TIST-enhanced expression pattern, ISH data, as well as IF data of the five marker genes *Adrab1, Ramp3, Enpp2, Klk8,* and *Hpca* are displayed. And typical patterns are emphasized in red boxes. **B.** SC identification test of Louvain (upper panel) and TIST (lower panel) for Ammon’s horn and dentate gyrus when diffusion rate *φ* is 0.2, 0.4, 0.6, 0.7, and 0.8. **C.** Comparison in the unsupervised modularity score Q to test clustering stability with diffusion rates between Louvain (blue line) and TIST (red line). Here, the X-axis shows the diffusion rate, and the Y-axis represents the classification accuracy. IF, immunofluorescence.

Supplementary Figure S6 **Supplementary results of dropout simulation of TIST A.** Recovery effects of TIST for four specific marker genes *Ramp3, Enpp2, Klk8,* and *Adrab1* as the DR increases from 0.1 to 0.8. **B.** Comparison of the unsupervised modularity score Q to test clustering stability with DRs between Louvain (blue line) and TIST (red line). Here, the X-axis shows the DR, and the Y-axis represents the modularity score Q.

Supplementary Figure S7 **Expression pattern****s****of top 100 SDEGs identified by TIST**

Supplementary Figure S8 **Expression pattern****s****of top 100 SDEGs identified by SPARK**

Supplementary Figure S9 **GO analysis of SDEGs detected by TIST and SPARK** Here, the X-axis represents the gene ratio, and the Y-axis represents GO terms in the categories BP (upper), CC (middle), MF (lower). The size of the bubble represents the number of enriched genes, and the colour represents the confidence level. GO, Gene Ontology; BP, biological process; CC, cellular component; MF, molecular function.

Supplementary Figure S10 **Comparison between TIST and stLearn on reliability of SDEGs A.** Gene recovery scores of TIST (left) and stLearn (right). The 49 marker genes related to brain activities are used. **B.** Comparison of DRs (left) and spatial expression pattern (right) between stLearn > TIST genes (stLearn achieves greater recovery score than TIST) and TIST > stLearn genes (TIST achieves greater recovery score than stLearn). *P* value is calculated by *t* test.

Supplementary Figure S11 **Benchmarking results of TIST on 8 public available datasets** SC identification results of TIST on public datasets from 10X. Each black box corresponds to one dataset. Figures on the left side within the boxes show the histopathological images. The right figures within the boxes show the automatic SC identification results of TIST

Supplementary Figure S12 **Benchmarking results of TIST on 20 human liver datasets of 10X** Each black box corresponds to a sample. The figures on the left side in the box show the results of the effective region and boundary outline of histopathological images. The right figures in the box show the SC identification results of TIST under the optimal parameter selection. The names of the samples are listed at the bottom of each box.

Supplementary Table S1 **Summary of all datasets utilized in this work**

Supplementary Table S2 **Region names in the mouse cerebral cortex from the reference atlas diagram from Allen Institute for Brain Science**

Supplementary Table S3 **Comparison among TIST and state-of-the-art SC identification methods**

Supplementary Table S4 **Recovery results of the 49 genes used for evaluation in Figure 4C**

Supplementary Table S5 **Previously published evidence of significant ligand-receptor pairs marked in Figure 6B**

## References

[1] Asp M., Bergenstråhle J., Lundeberg J. (2020). Spatially resolved transcriptomes — next generation tools for tissue exploration. Bioessays.

[2] Eng C.H.L., Lawson M., Zhu Q., Dries R., Koulena N., Takei Y. (2019). Transcriptome-scale super-resolved imaging in tissues by RNA seqFISH+. Nature.

[3] Rodriques S.G., Stickels R.R., Goeva A., Martin C.A., Murray E., Vanderburg C.R. (2019). Slide-seq: a scalable technology for measuring genome-wide expression at high spatial resolution. Science.

[4] Cho C.S., Xi J., Si Y., Park S.R., Hsu J.E., Kim M. (2021). Microscopic examination of spatial transcriptome using Seq-Scope. Cell.

[5] Chen A., Liao S., Cheng M., Ma K., Wu L., Lai Y. (2022). Spatiotemporal transcriptomic atlas of mouse organogenesis using DNA nanoball-patterned arrays. Cell.

[6] Blondel V.D., Guillaume J.L., Lambiotte R., Lefebvre E. (2008). Fast unfolding of communities in large networks. J Stat Mech Theory Exp.

[7] Hao Y., Hao S., Andersen-Nissen E., Mauck W.M., Zheng S., Butler A. (2021). Integrated analysis of multimodal single-cell data. Cell.

[8] Zhao E., Stone M.R., Ren X., Guenthoer J., Smythe K.S., Pulliam T. (2021). Spatial transcriptomics at subspot resolution with BayesSpace. Nat Biotechnol.

[9] Dong K., Zhang S. (2022). Deciphering spatial domains from spatially resolved transcriptomics with an adaptive graph attention auto-encoder. Nat Commun.

[10] Chen Y, Zhou S, Li M, Zhao F, Qi J. STEEL enables high-resolution delineation of spatiotemporal transcriptomic data. Research Square 2022. https://doi.org/10.21203/rs.3.rs-1240258/v1. 10.1093/bib/bbad068 36857617

[11] Fu H, Xu H, Chong K, Li M, Ang KS, Lee HK (2021). Unsupervised spatially embedded deep representation of spatial transcriptomics. bioRxiv.

[12] Sun S., Zhu J., Zhou X. (2020). Statistical analysis of spatial expression patterns for spatially resolved transcriptomic studies. Nat Methods.

[13] Pham D, Tan X, Xu J, Grice LF, Lam PY, Raghubar A (2020). stLearn: integrating spatial location, tissue morphology and gene expression to find cell types, cell–cell interactions and spatial trajectories within undissociated tissues. bioRxiv.

[14] Hu J., Li X.J., Coleman K., Schroeder A., Ma N., Irwin D.J. (2021). SpaGCN: integrating gene expression, spatial location and histology to identify spatial domains and spatially variable genes by graph convolutional network. Nat Methods.

[15] Cutiongco M.F.A., Jensen B.S., Reynolds P.M., Gadegaard N. (2020). Predicting gene expression using morphological cell responses to nanotopography. Nat Commun.

[16] Jarvis R.A., Patrick E.A. (1973). Clustering using a similarity measure based on shared near neighbors. IEEE Trans Comput.

[17] Li S.Z. (2003). Modeling image analysis problems using Markov random fields. Handb Stat.

[18] Pons P., Latapy M., Yolum P., Güngör T., Gürgen F., Özturan C. (2005, p.284–93). Computer and Information Sciences - ISCIS 2005.

[19] Venable R.M., Kramer A., Pastor R.W. (2019). Molecular dynamics simulations of membrane permeability. Chem Rev.

[20] Newman M.E.J. (2006). Modularity and community structure in networks. Proc Natl Acad Sci U S A.

[21] Wu R., Guo W., Qiu X., Wang S., Sui C., Lian Q. (2021). Comprehensive analysis of spatial architecture in primary liver cancer. Sci Adv.

[22] Wang Q., Ding S.L., Li Y., Royall J., Feng D., Lesnar P. (2020). The Allen Mouse Brain Common Coordinate Framework: a 3D reference atlas. Cell.

[23] Zeidman P., Maguire E.A. (2016). Anterior hippocampus: the anatomy of perception, imagination and episodic memory. Nat Rev Neurosci.

[24] Strange B.A., Witter M.P., Lein E.S., Moser E.I. (2014). Functional organization of the hippocampal longitudinal axis. Nat Rev Neurosci.

[25] Helmink B.A., Reddy S.M., Gao J., Zhang S., Basar R., Thakur R. (2020). B cells and tertiary lymphoid structures promote immunotherapy response. Nature.

[26] Sautes-Fridman C., Petitprez F., Calderaro J., Fridman W.H. (2019). Tertiary lymphoid structures in the era of cancer immunotherapy. Nat Rev Cancer.

[27] Tokunaga R., Nakagawa S., Sakamoto Y., Nakamura K., Naseem M., Izumi D. (2020). 12-chemokine signature, a predictor of tumor recurrence in colorectal cancer. Int J Cancer.

[28] Galeano F., Rossetti C., Tomaselli S., Cifaldi L., Lezzerini M., Pezzullo M. (2013). ADAR2-editing activity inhibits glioblastoma growth through the modulation of the CDC14B/Skp2/p21/p27 axis. Oncogene.

[29] Zembrzycki A., Stocker A.M., Leingartner A., Sahara S., Chou S.J., Kalatsky V. (2015). Genetic mechanisms control the linear scaling between related cortical primary and higher order sensory areas. Elife.

[30] Jin S.Q., Guerrero-Juarez C.F., Zhang L.H., Chang I., Ramos R., Kuan C.H. (2021). Inference and analysis of cell–cell communication using Cell Chat. Nat Commun.

[31] Arredondo S.B., Valenzuela-Bezanilla D., Mardones M.D., Varela-Nallar L. (2020). Role of Wnt signaling in adult hippocampal neurogenesis in health and disease. Front Cell Dev Biol.

[32] Bonifazi P., Goldin M., Picardo M.A., Jorquera I., Cattani A., Bianconi G. (2009). GABAergic hub neurons orchestrate synchrony in developing hippocampal networks. Science.

[33] Rao A., Barkley D., Franca G.S., Yanai I. (2021). Exploring tissue architecture using spatial transcriptomics. Nature.

