# Supplementary figures and images for: TIST: Transcriptome and Histopathological Image Integrative Analysis for Spatial Transcriptomics
Source: Genomics Proteomics Bioinformatics. 2022 Dec 19;20(5):974–88. doi: 10.1016/j.gpb.2022.11.012 (PMC10025771; doi:10.1016/j.gpb.2022.11.012)

A

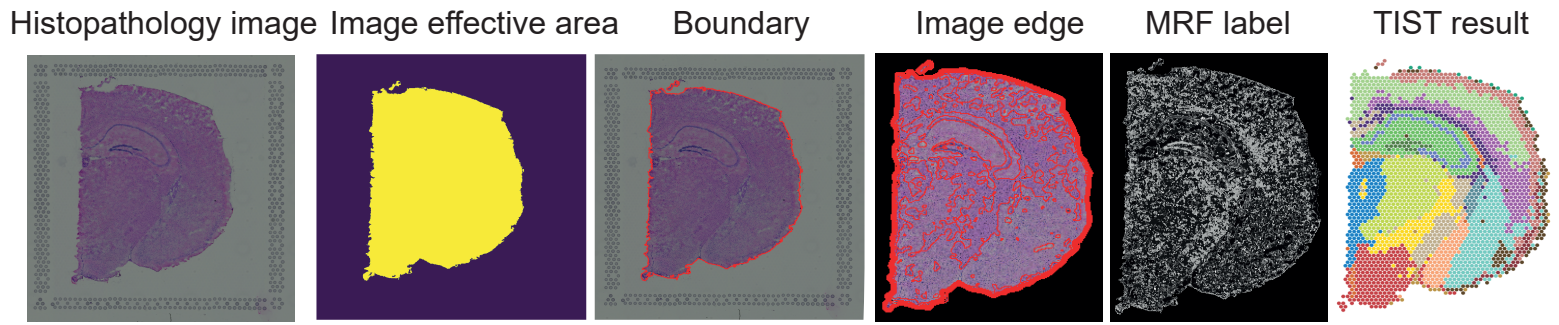

B

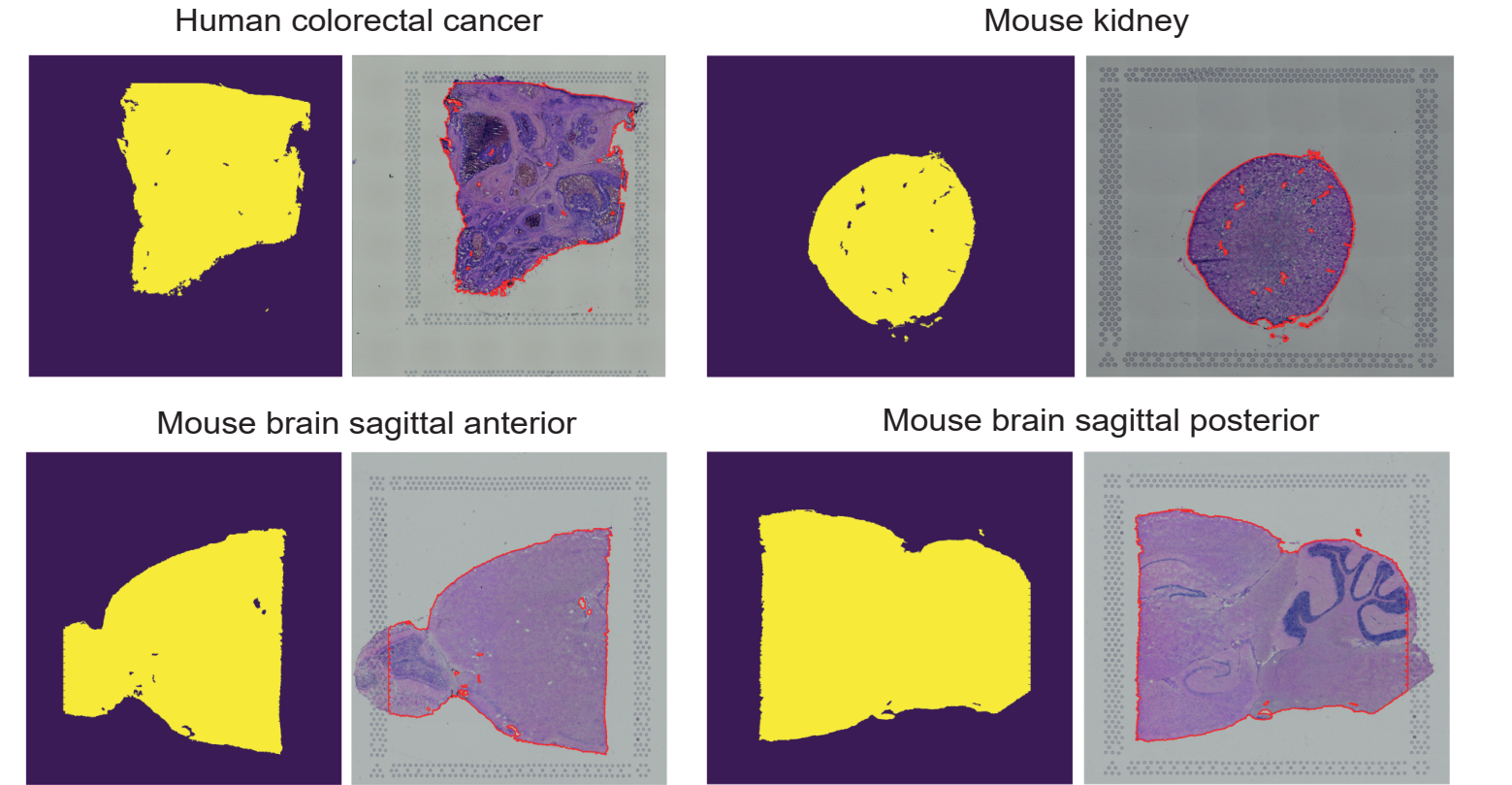

Supplement: Supplementary Figure S1 — Processing flow of TIST A. Typical TIST processing on histopathology image. For the original image, we first obtain the outer boundary outline and explore the whole image to obtain inner lines. Boundary and edge information is then obtained. We use MRF to label this image and implement TIST to further recognize SCs. B. TIST process on the histopathology image of human colorectal cancer (upper left), mouse kidney (upper right), mouse brain sagittal anterior (lower left), mouse brain sagittal posterior (lower right) datasets. MRF, Markov random field. [file mmc1.pdf]

A

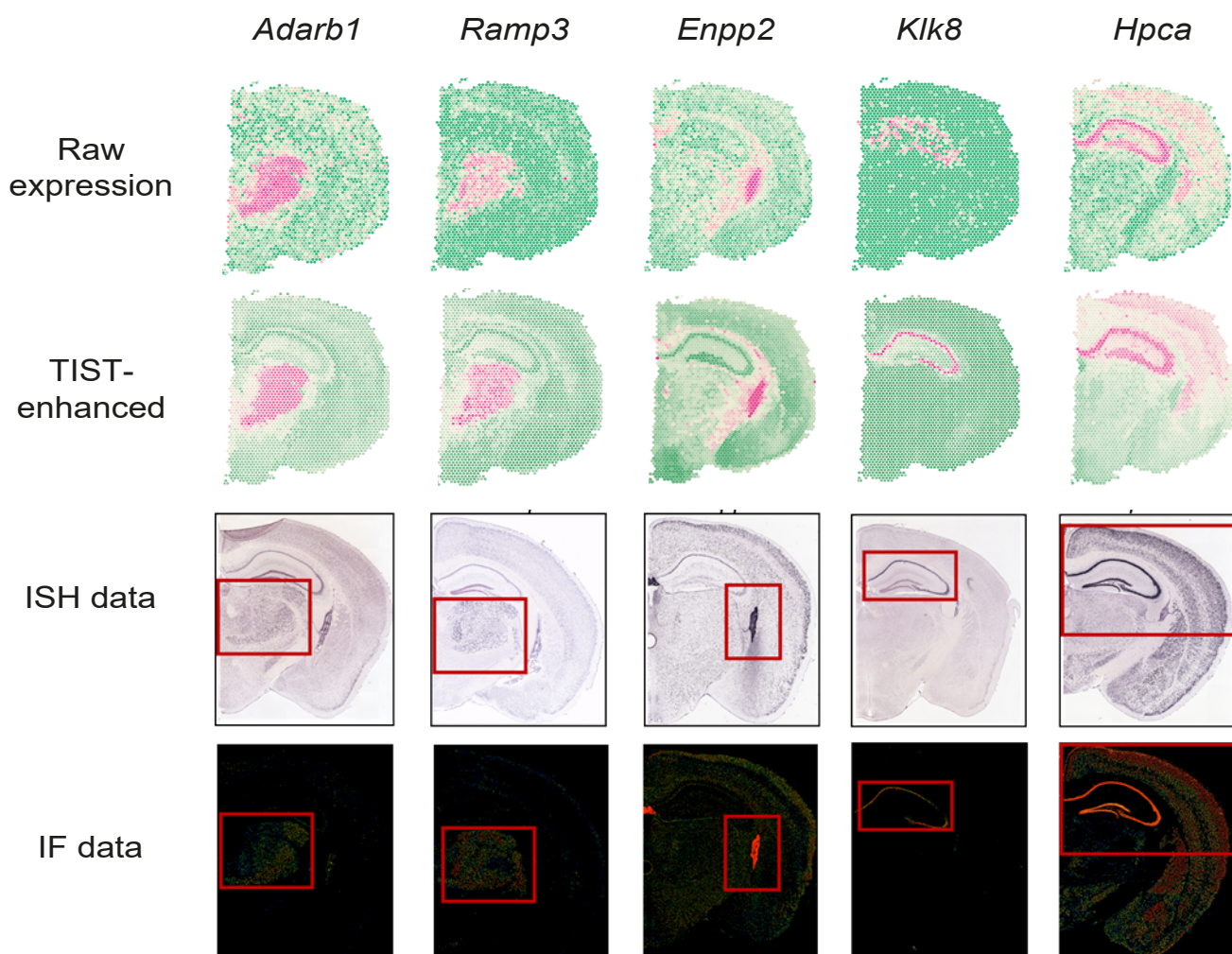

B

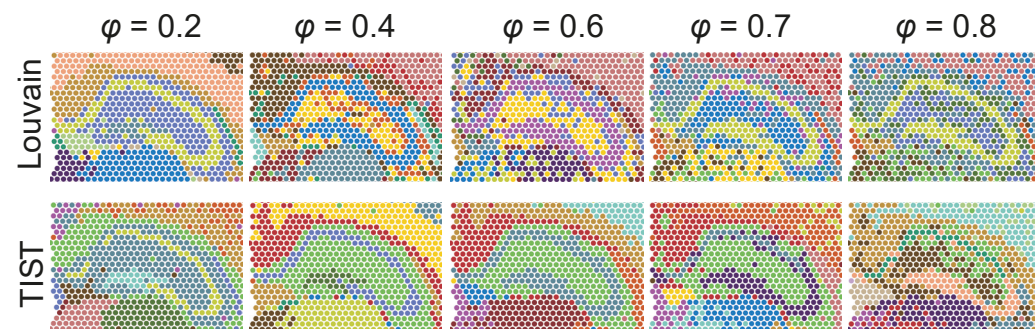

C

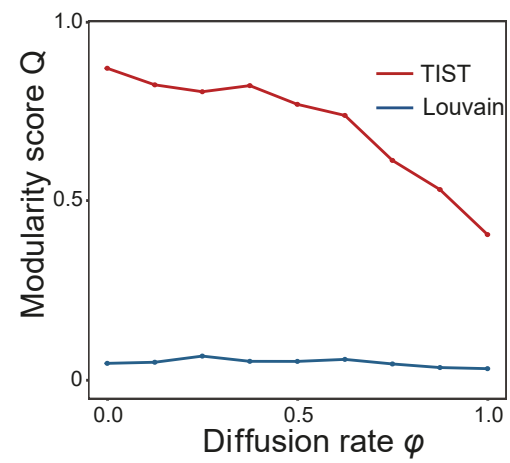

Supplement: Supplementary Figure S5 — Expression patterns of typical marker genes and comparison between TIST and Louvain in diffusion simulation A. Raw expression, TIST-enhanced expression pattern, ISH data, as well as IF data of the five marker genes Adrab1, Ramp3, Enpp2, Klk8, and Hpca are displayed. And typical patterns are emphasized in red boxes. B. SC identification test of Louvain (upper panel) and TIST (lower panel) for Ammon’s horn and dentate gyrus when diffusion rate φ is 0.2, 0.4, 0.6, 0.7, and 0.8. C. Comparison in the unsupervised modularity score Q to test clustering stability with diffusion rates between Louvain (blue line) and TIST (red line). Here, the X-axis shows the diffusion rate, and the Y-axis represents the classification accuracy. IF, immunofluorescence. [file mmc5.pdf]

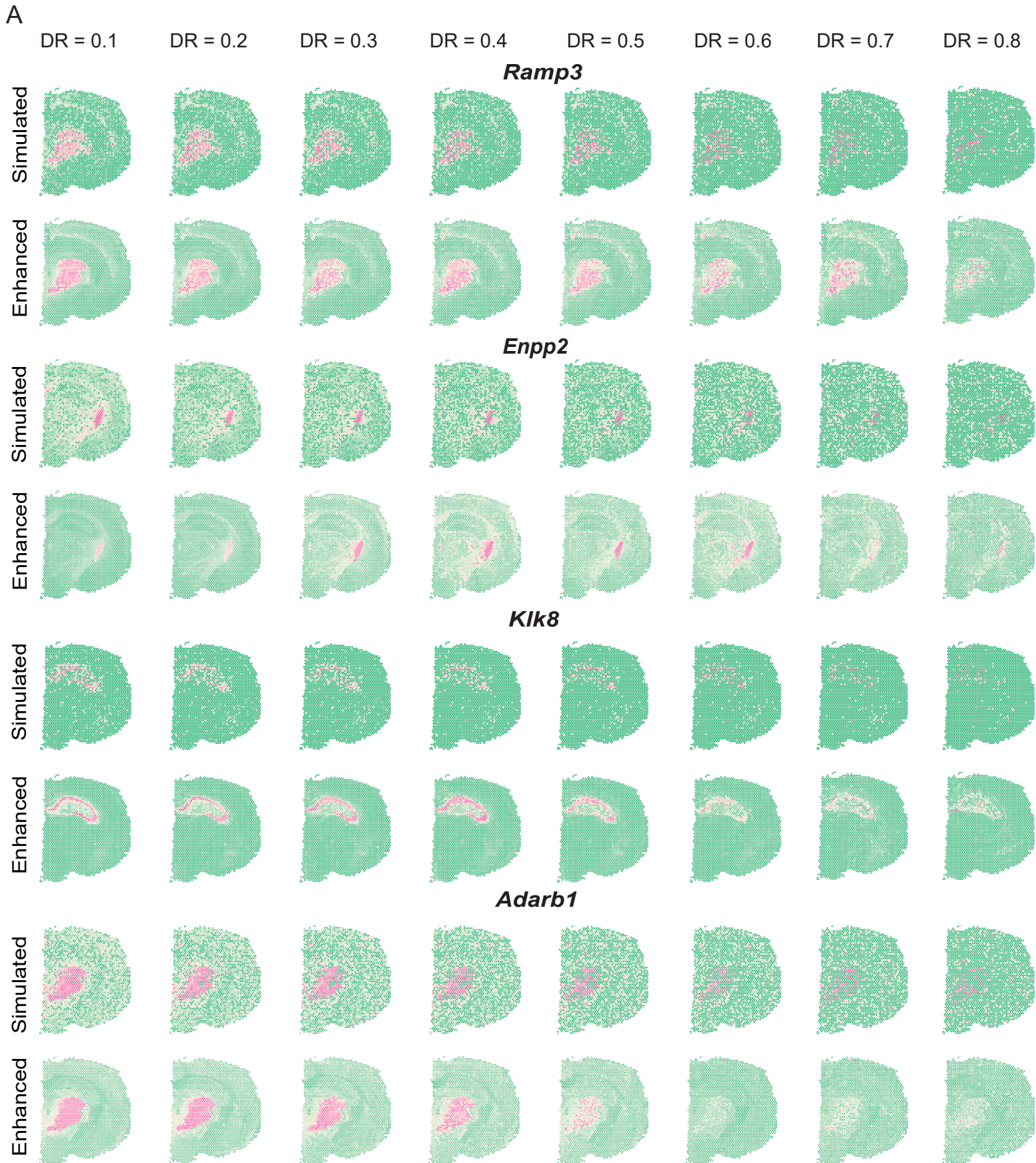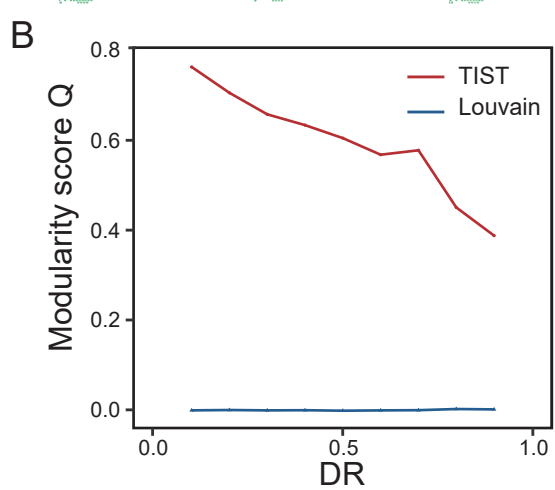

Supplement: Supplementary Figure S6 — Supplementary results of dropout simulation of TIST A. Recovery effects of TIST for four specific marker genes Ramp3, Enpp2, Klk8, and Adrab1 as the DR increases from 0.1 to 0.8. B. Comparison of the unsupervised modularity score Q to test clustering stability with DRs between Louvain (blue line) and TIST (red line). Here, the X-axis shows the DR, and the Y-axis represents the modularity score Q. [file mmc6.pdf]

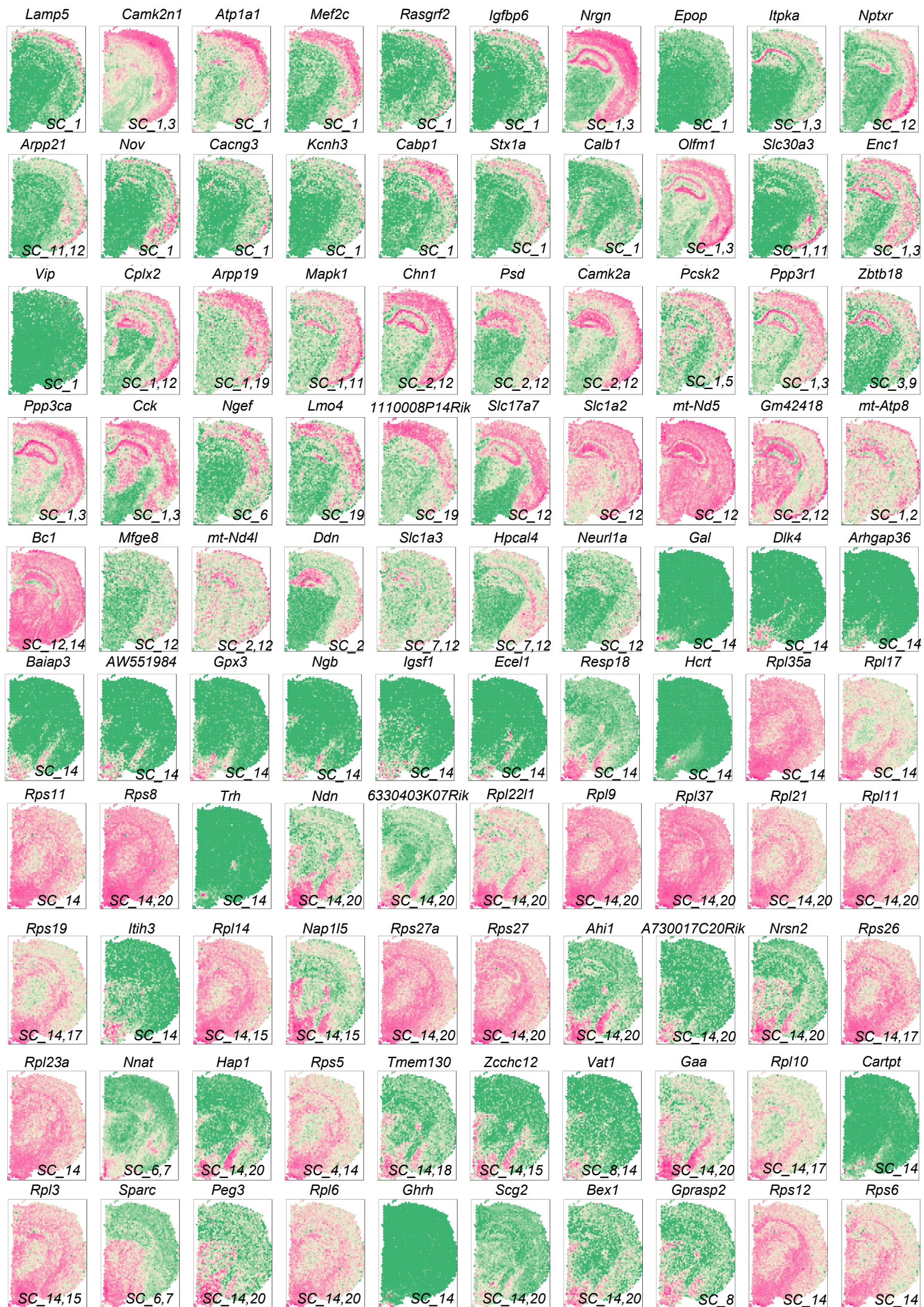

Supplement: Supplementary Figure S7 — Expression patternsof top 100 SDEGs identified by TIST [file mmc7.pdf]

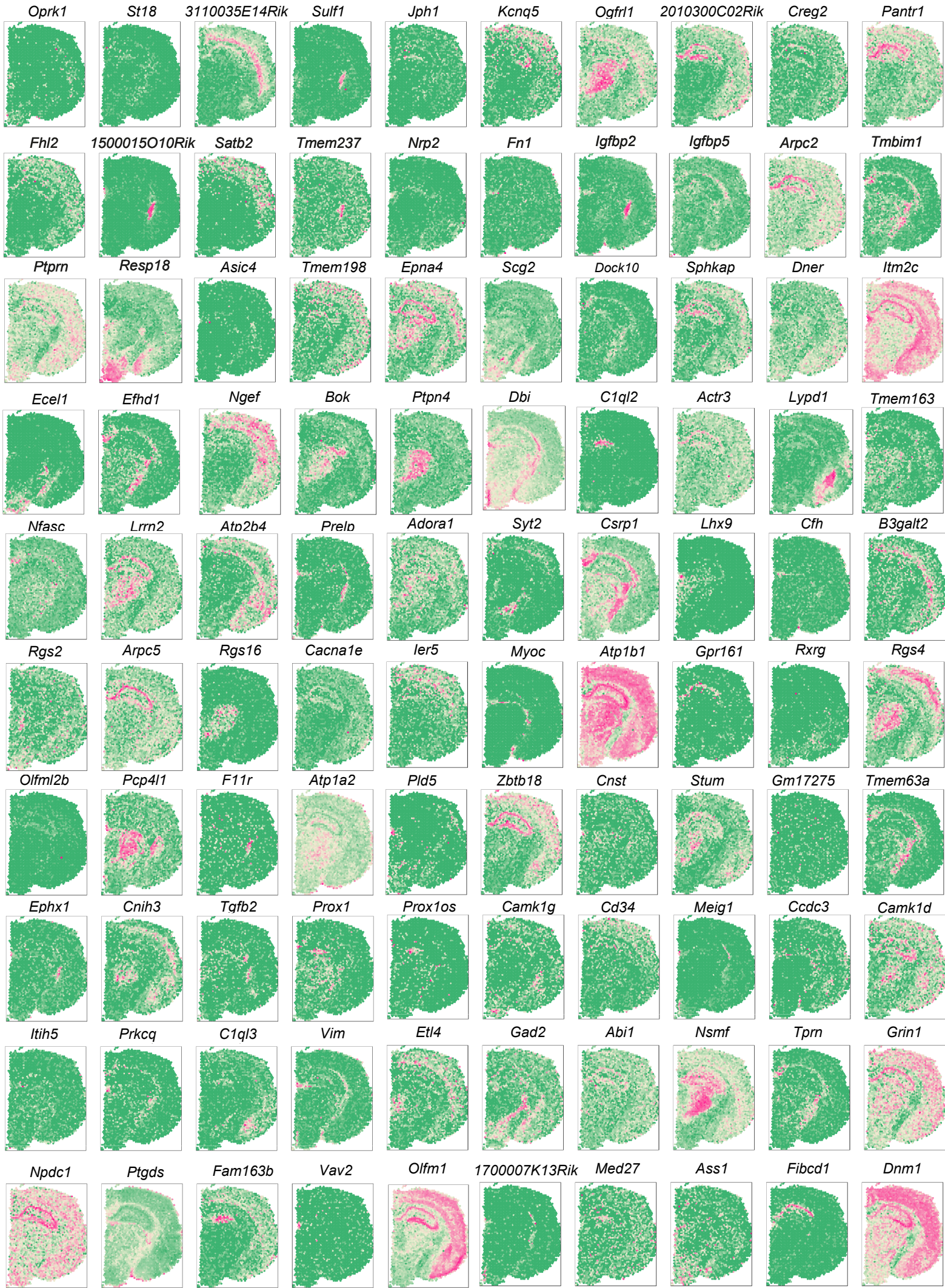

Supplement: Supplementary Figure S8 — Expression patternsof top 100 SDEGs identified by SPARK [file mmc8.pdf]

## TIST

## SPARK

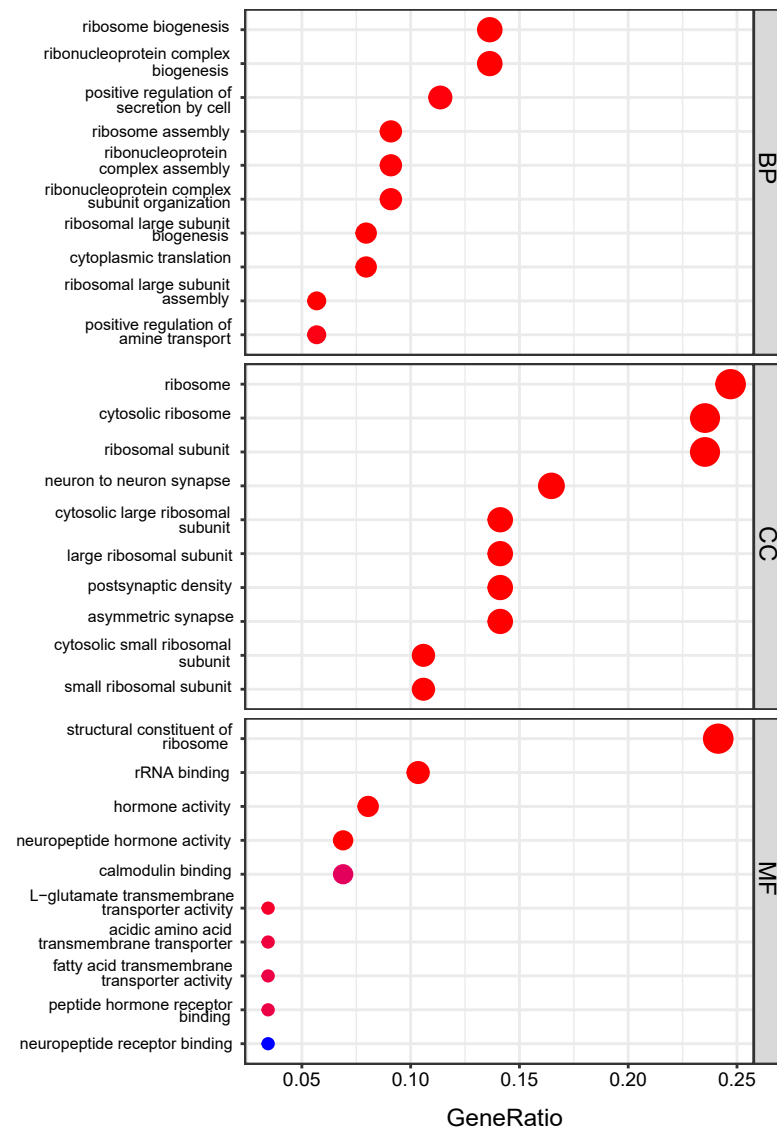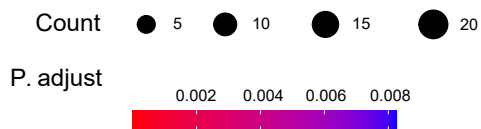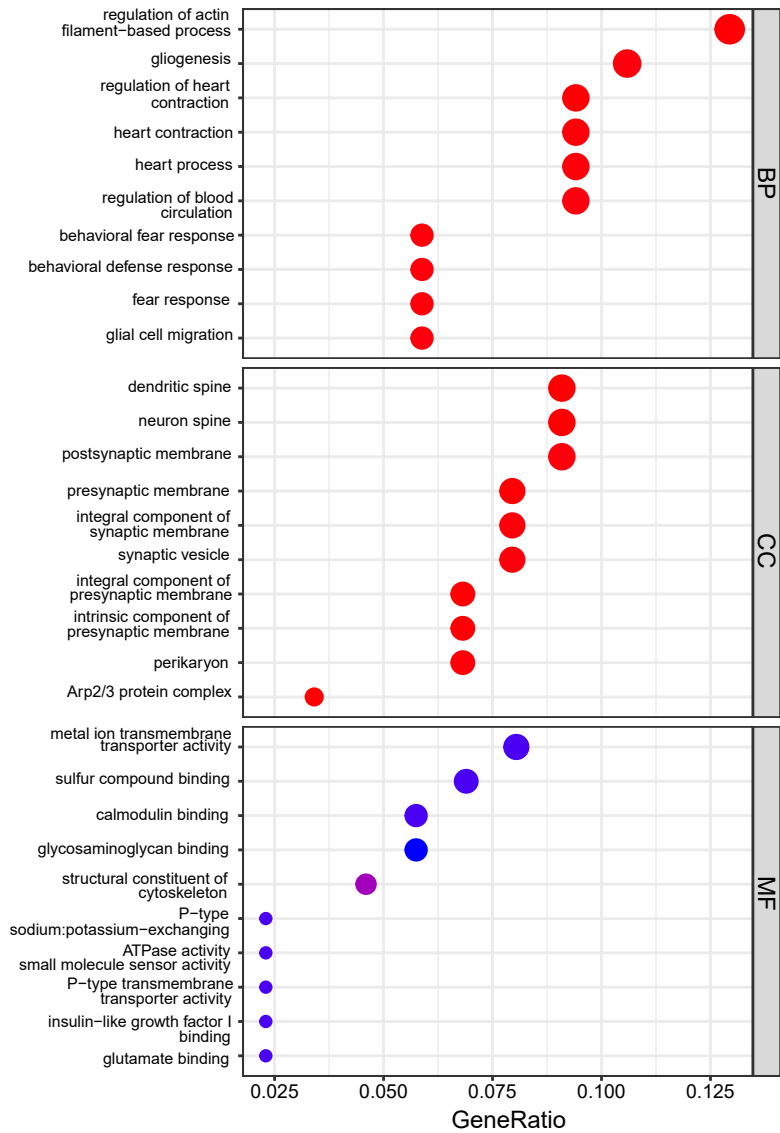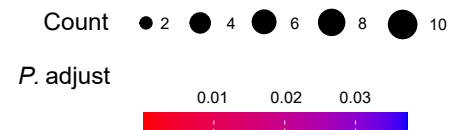

Supplement: Supplementary Figure S9 — GO analysis of SDEGs detected by TIST and SPARK Here, the X-axis represents the gene ratio, and the Y-axis represents GO terms in the categories BP (upper), CC (middle), MF (lower). The size of the bubble represents the number of enriched genes, and the colour represents the confidence level. GO, Gene Ontology; BP, biological process; CC, cellular component; MF, molecular function. [file mmc9.pdf]

A

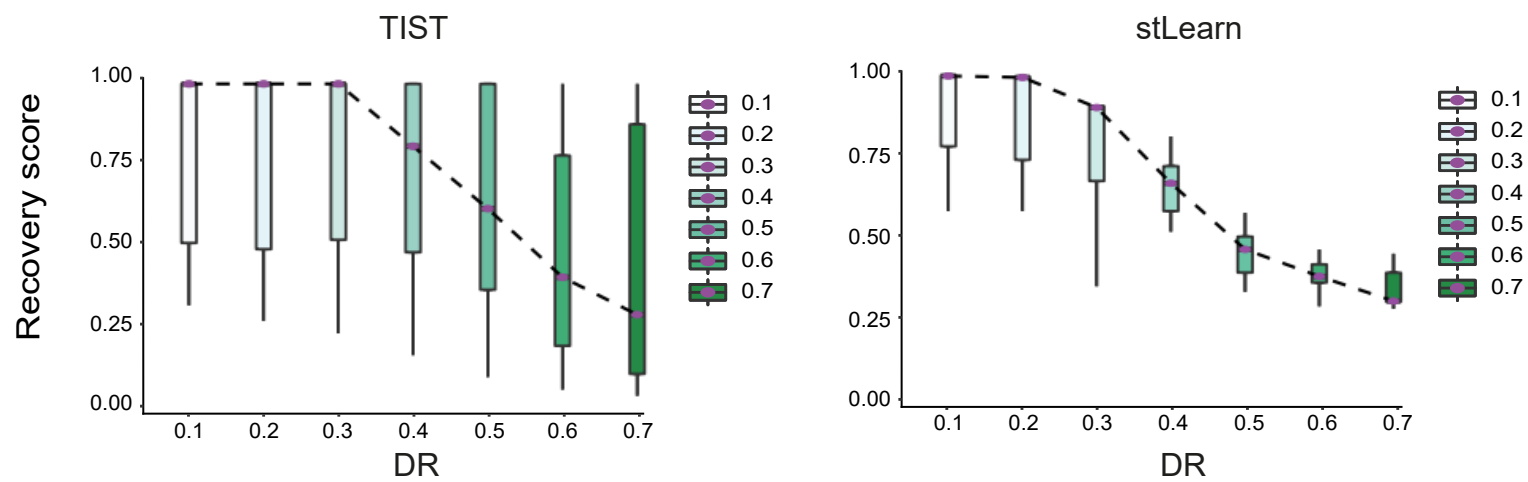

B

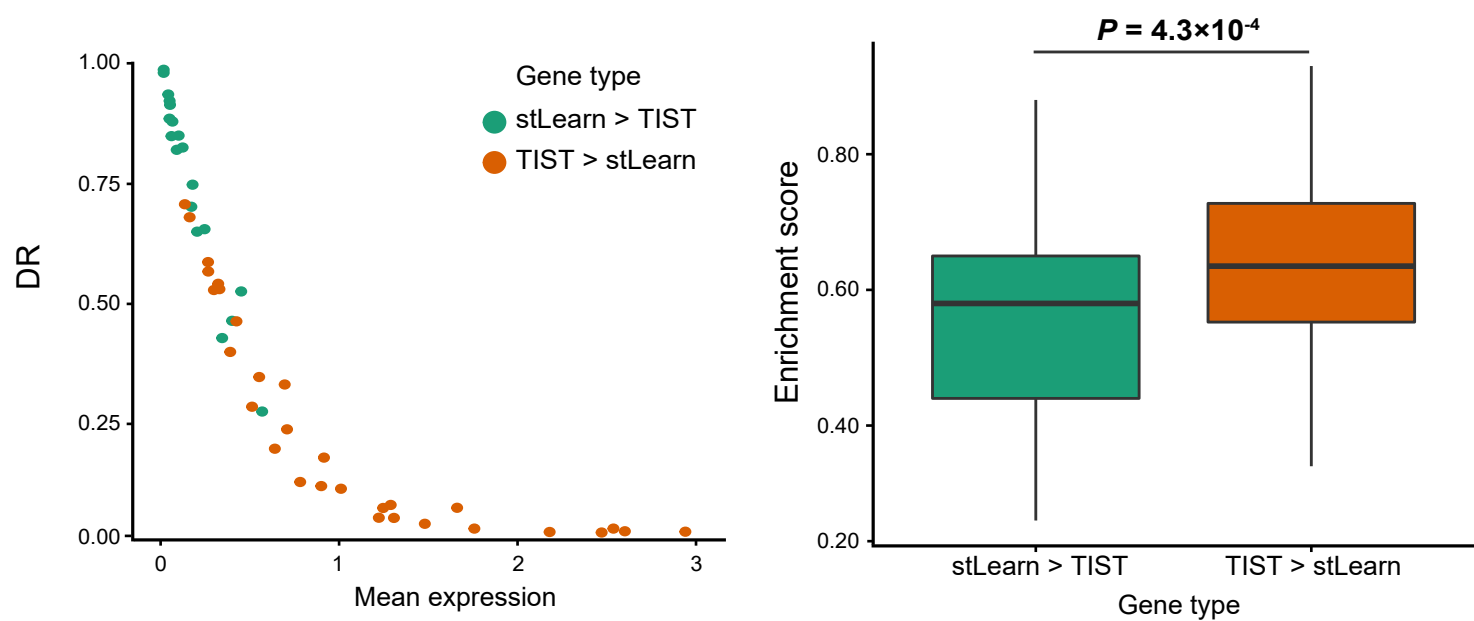

Supplement: Supplementary Figure S10 — Comparison between TIST and stLearn on reliability of SDEGs A. Gene recovery scores of TIST (left) and stLearn (right). The 49 marker genes related to brain activities are used. B. Comparison of DRs (left) and spatial expression pattern (right) between stLearn > TIST genes (stLearn achieves greater recovery score than TIST) and TIST > stLearn genes (TIST achieves greater recovery score than stLearn). P value is calculated by t test. [file mmc10.pdf]

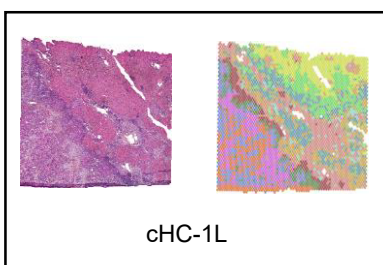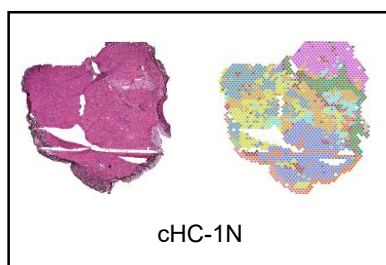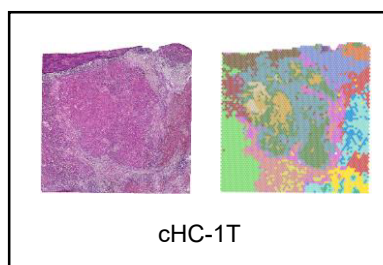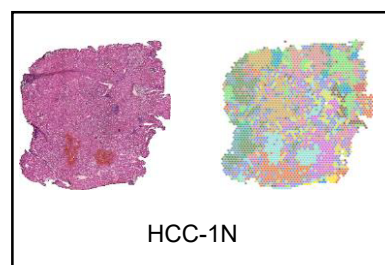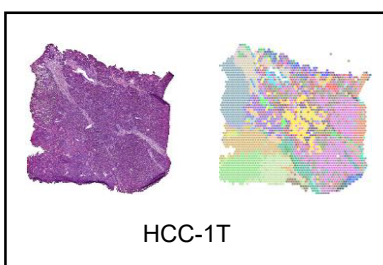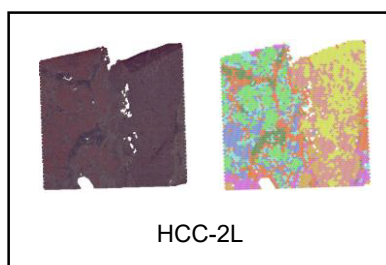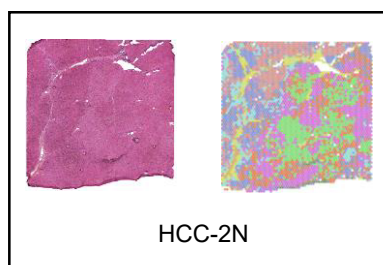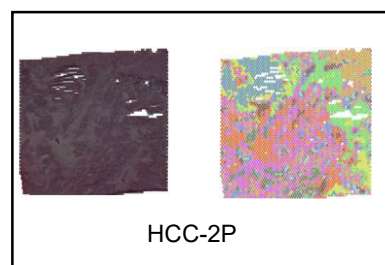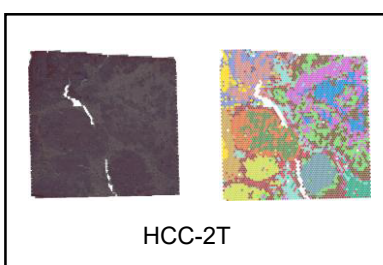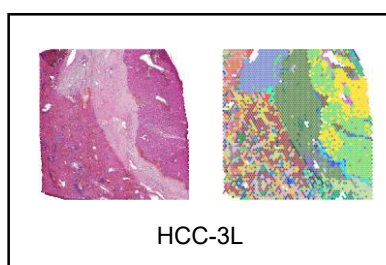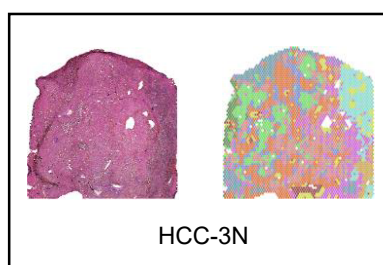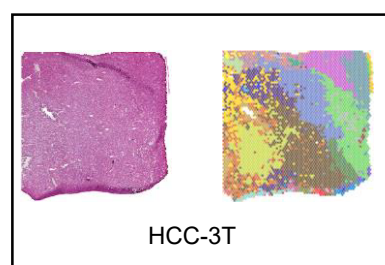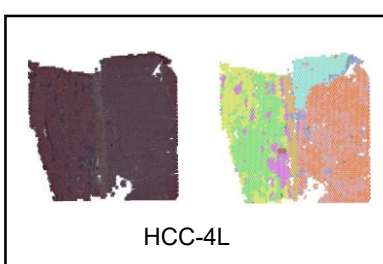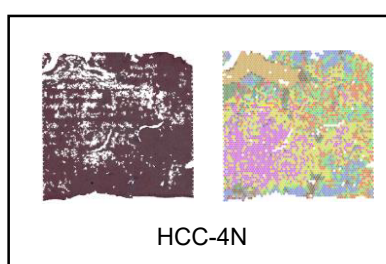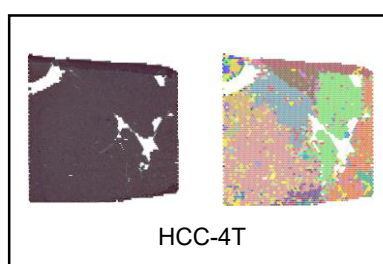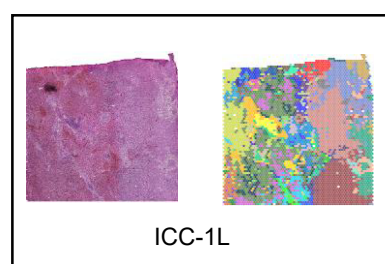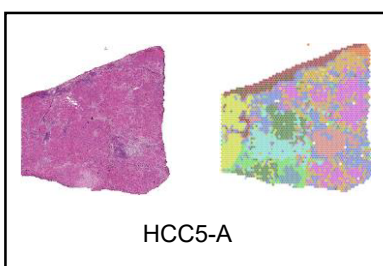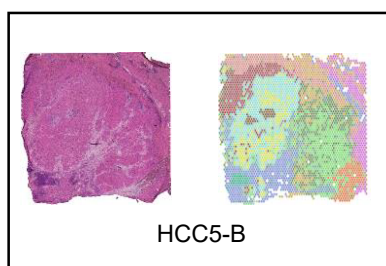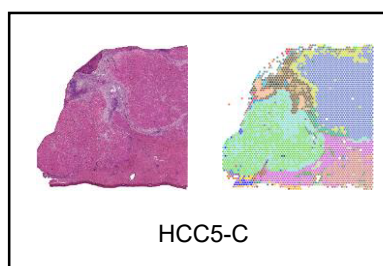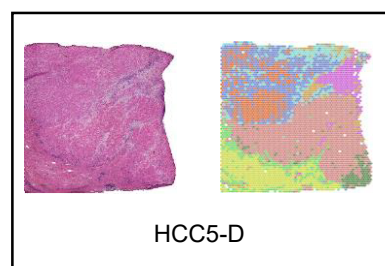

Supplement: Supplementary Figure S12 — Benchmarking results of TIST on 20 human liver datasets of 10X Each black box corresponds to a sample. The figures on the left side in the box show the results of the effective region and boundary outline of histopathological images. The right figures in the box show the SC identification results of TIST under the optimal parameter selection. The names of the samples are listed at the bottom of each box. [file mmc12.pdf]
