# Supplementary material for: TIST: Transcriptome and Histopathological Image Integrative Analysis for Spatial Transcriptomics
Source: Genomics Proteomics Bioinformatics. 2022 Dec 19;20(5):974–88. doi: 10.1016/j.gpb.2022.11.012 (PMC10025771; doi:10.1016/j.gpb.2022.11.012)
Supplement: Supplementary Figure S2 — Comparison of Louvain and TIST on the mouse cerebral cortex data A. Histopathological image from 10X Visium platform and detailed manual annotation for predominant structures of mouse cerebral cortex from Allen Mouse Brain Atlas. B. SC identification results of Louvain at the best performance resolution 0.3 displayed over the spatial locations (left) and UMAP-transformed 2D pane (right). C. SC identification results of TIST displayed over the spatial locations (left) and the UMAP-transformed 2D pane (right). D. SC identification of Louvain with resolution adjusted in the range 0.1 to 2.0. The number of SCs is shown in the figure, while the clustering effect quantified by ARI is also shown. UMAP, uniform manifold approximation and projection; 2D, 2 dimensional. [file mmc2.pdf]

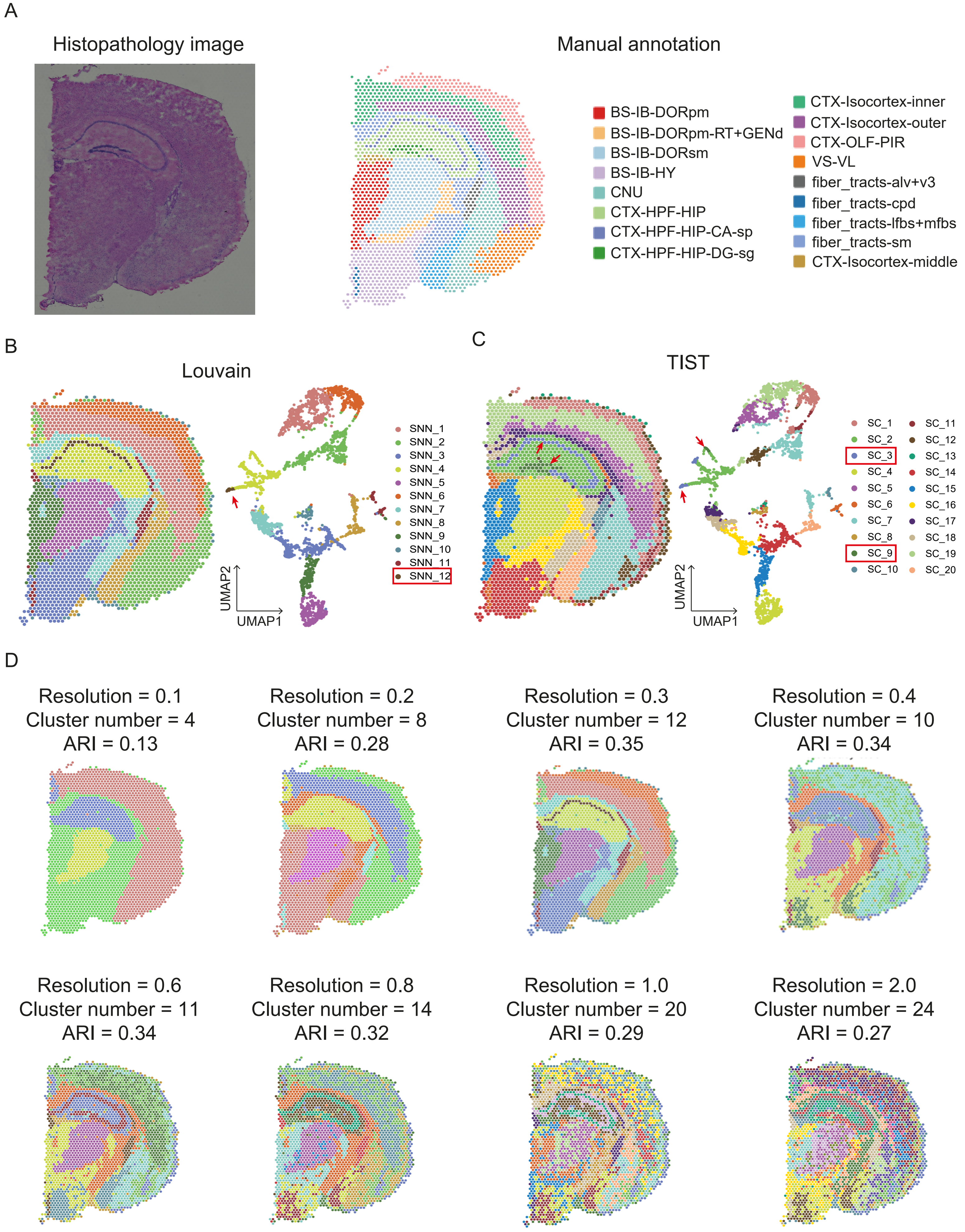
A
Histopathology image
Manual annotation
BS-IB-DORpm
BS-IB-DORpm-RT+GENd
BS-IB-DORsm
BS-IB-HY
CNU
CTX-HPF-HIP
CTX-HPF-HIP-CA-sp
CTX-HPF-HIP-DG-sg
CTX-Isocortex-inner
CTX-Isocortex-outer
CTX-OLF-PIR
VS-VL
fiber_tracts-alv+v3
fiber_tracts-cpd
fiber_tracts-lfbs+mfbs
fiber_tracts-sm
CTX-Isocortex-middle
B
Louvain
UMAP2
UMAP1
SNN_1
SNN_2
SNN_3
SNN_4
SNN_5
SNN_6
SNN_7
SNN_8
SNN_9
SNN_10
SNN_11
SNN_12
C
TIST
UMAP2
UMAP1
SC_1
SC_2
SC_3
SC_4
SC_5
SC_6
SC_7
SC_8
SC_9
SC_10
SC_11
SC_12
SC_13
SC_14
SC_15
SC_16
SC_17
SC_18
SC_19
SC_20
D
Resolution = 0.1
Cluster number = 4
ARI = 0.13
Resolution = 0.2
Cluster number = 8
ARI = 0.28
Resolution = 0.3
Cluster number = 12
ARI = 0.35
Resolution = 0.4
Cluster number = 10
ARI = 0.34
Resolution = 0.6
Cluster number = 11
ARI = 0.34
Resolution = 0.8
Cluster number = 14
ARI = 0.32
Resolution = 1.0
Cluster number = 20
ARI = 0.29
Resolution = 2.0
Cluster number = 24
ARI = 0.27
