# Supplementary material for: TIST: Transcriptome and Histopathological Image Integrative Analysis for Spatial Transcriptomics
Source: Genomics Proteomics Bioinformatics. 2022 Dec 19;20(5):974–88. doi: 10.1016/j.gpb.2022.11.012 (PMC10025771; doi:10.1016/j.gpb.2022.11.012)
Supplement: Supplementary Figure S3 — Ablation experiment of TIST Each component of TIST-net (three networks of histopathological information, transcriptomic information, and physical information) is removed once time, and TIST is then utilized on the mouse cerebral cortex dataset. Identification accuracy metrics of ARI and NMI are listed. [file mmc3.pdf]

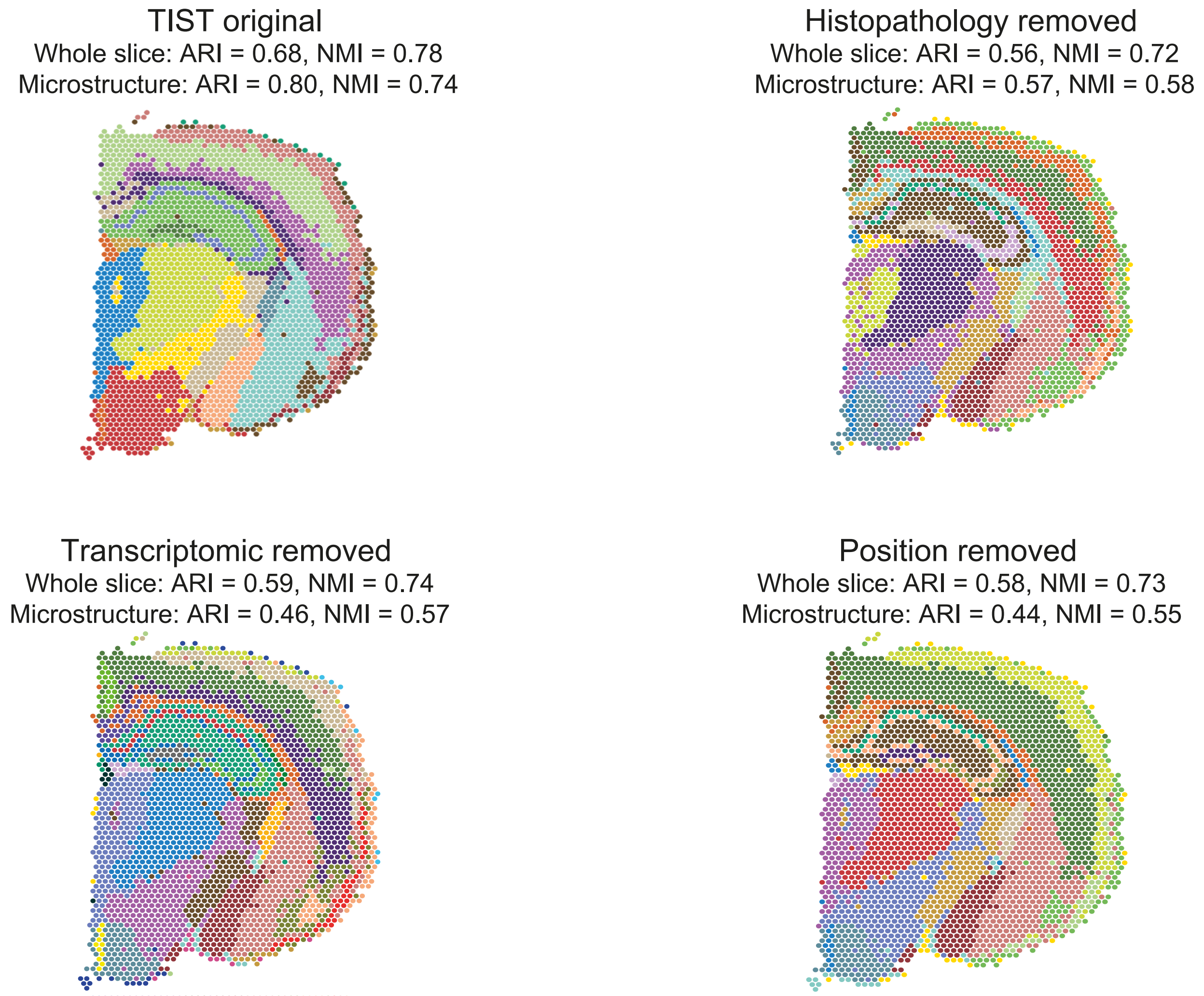
TIST original
Whole slice: ARI = 0.68, NMI = 0.78
Microstructure: ARI = 0.80, NMI = 0.74
Histopathology removed
Whole slice: ARI = 0.56, NMI = 0.72
Microstructure: ARI = 0.57, NMI = 0.58
Transcriptomic removed
Whole slice: ARI = 0.59, NMI = 0.74
Microstructure: ARI = 0.46, NMI = 0.57
Position removed
Whole slice: ARI = 0.58, NMI = 0.73
Microstructure: ARI = 0.44, NMI = 0.55
