# Supplementary material for: TIST: Transcriptome and Histopathological Image Integrative Analysis for Spatial Transcriptomics
Source: Genomics Proteomics Bioinformatics. 2022 Dec 19;20(5):974–88. doi: 10.1016/j.gpb.2022.11.012 (PMC10025771; doi:10.1016/j.gpb.2022.11.012)
Supplement: Supplementary Figure S4 — Supplementary results of diffusion simulation of TIST A. Test of TIST gene enhancement for Adarb1 when diffusion rate φ is from 0.1 to 0.9 excluding exhibited in Figure 3C, respectively. B. Spatial expression patterns of the specific marker genes Ramp3, Enpp2, Klk8, Hpca, Cabp7, and Hap1 when setting the diffusion rate φ as 0.5. [file mmc4.pdf]

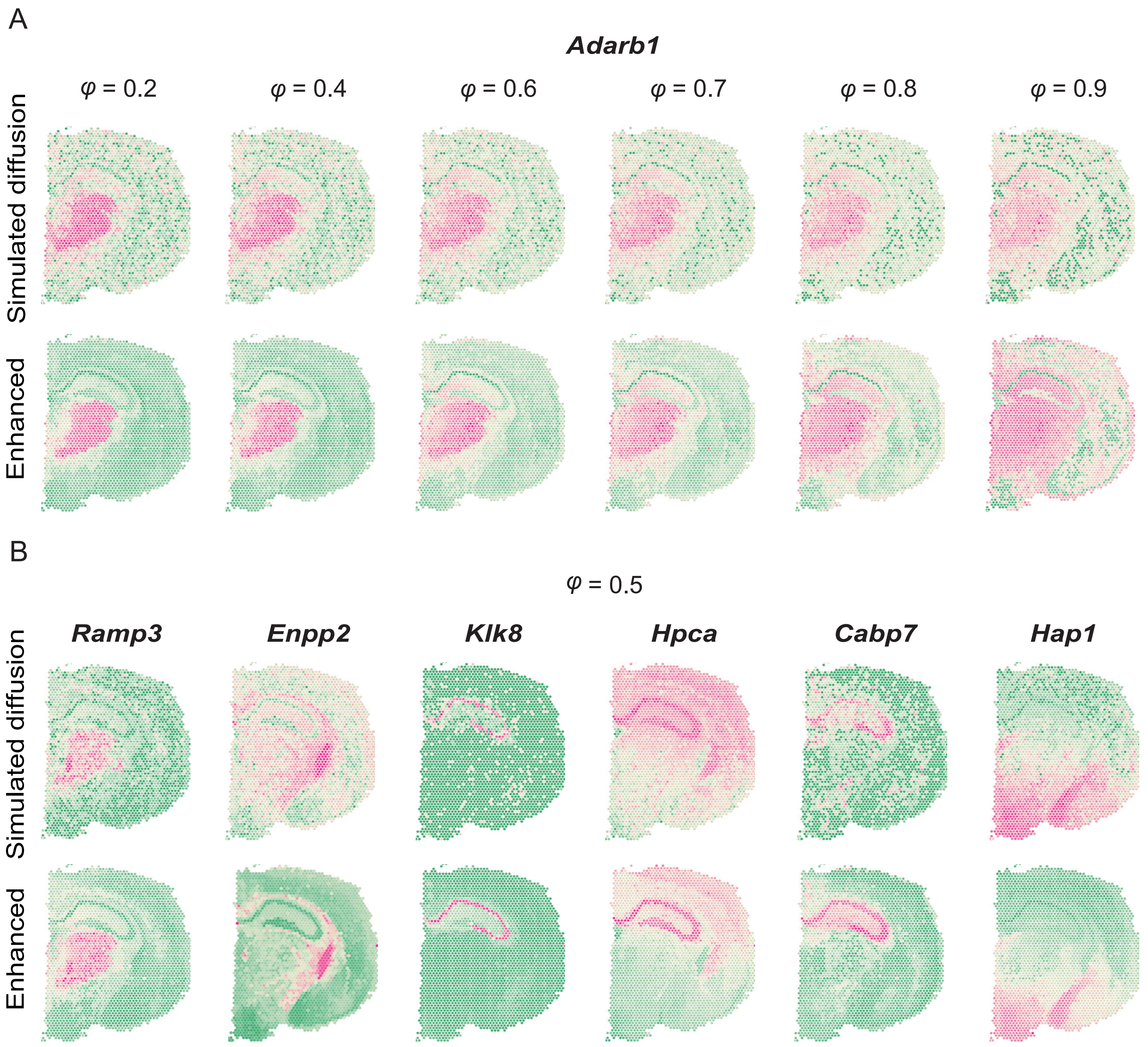
A
Adarb1
φ = 0.2
φ = 0.4
φ = 0.6
φ = 0.7
φ = 0.8
φ = 0.9
Simulated diffusion
Enhanced
B
φ = 0.5
Ramp3
Enpp2
Klk8
Hpca
Cabp7
Hap1
Simulated diffusion
Enhanced
