# Supplementary material for: TIST: Transcriptome and Histopathological Image Integrative Analysis for Spatial Transcriptomics
Source: Genomics Proteomics Bioinformatics. 2022 Dec 19;20(5):974–88. doi: 10.1016/j.gpb.2022.11.012 (PMC10025771; doi:10.1016/j.gpb.2022.11.012)
Supplement: Supplementary Figure S11 — Benchmarking results of TIST on 8 public available datasets SC identification results of TIST on public datasets from 10X. Each black box corresponds to one dataset. Figures on the left side within the boxes show the histopathological images. The right figures within the boxes show the automatic SC identification results of TIST [file mmc11.pdf]

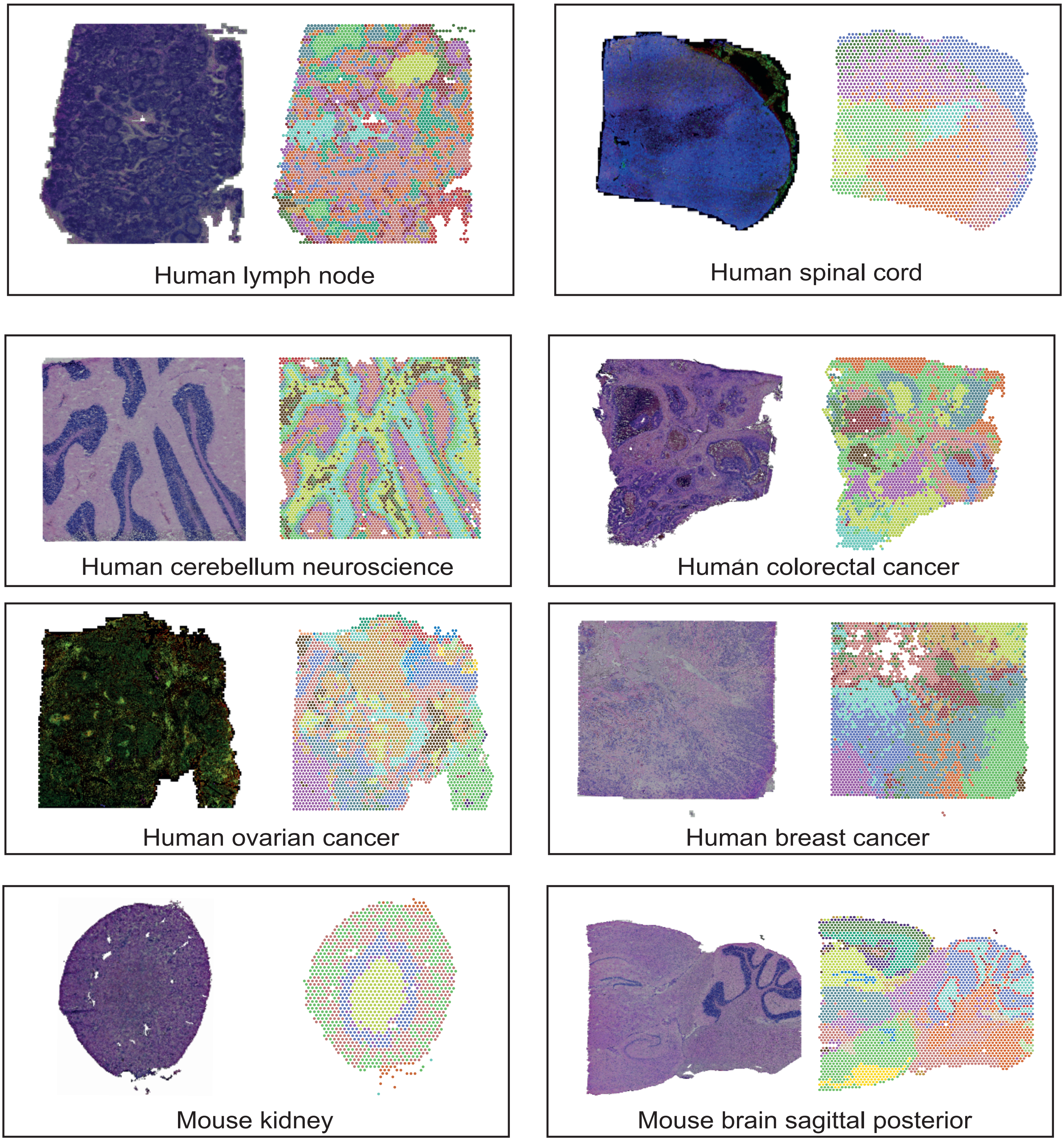

Human lymph node
Human spinal cord
Human cerebellum neuroscience
Humán colorectal cancer
Human ovarian cancer
Human breast cancer
Mouse kidney
Mouse brain sagittal posterior
