# Supplementary material for: TIST: Transcriptome and Histopathological Image Integrative Analysis for Spatial Transcriptomics
Source: Genomics Proteomics Bioinformatics. 2022 Dec 19;20(5):974–88. doi: 10.1016/j.gpb.2022.11.012 (PMC10025771; doi:10.1016/j.gpb.2022.11.012)
Supplement: Supplementary Table S1 — Summary of all datasets utilized in this work [file mmc13.docx]

**Table S1 Summary of all datasets utilized in this work**

| **Sample** | **Species** | **Percentage of UMI counts in blank area (%)** | **Median No. of genes per spot** | **Number of tissue-covered spots** | **Data resource** |
| --- | --- | --- | --- | --- | --- |
| Human breast cancer | Human | 3.74 | 3671 | 4169 | 10x Genomics (https://www.10xgenomics.com/resources/datasets) |
| Breast cancer block A section 1 | Human | 6.29 | 5394 | 3813 | 10x Genomics (https://www.10xgenomics.com/resources/datasets) |
| Human lymph node | Human | 10.58 | 5347 | 4033 | 10x Genomics (https://www.10xgenomics.com/resources/datasets) |
| Human spinal cord | Human | 15.13 | 2253 | 2809 | 10x Genomics (https://www.10xgenomics.com/resources/datasets) |
| Human cerebellum neuroscience | Human | 6.75 | 2865 | 4916 | 10x Genomics (https://www.10xgenomics.com/resources/datasets) |
| Human colorectal cancer | Human | 23.60 | 3538 | 3137 | 10x Genomics (https://www.10xgenomics.com/resources/datasets) |
| Human ovarian cancer | Human | 16.75 | 3464 | 3489 | 10x Genomics (https://www.10xgenomics.com/resources/datasets) |
| Adult mouse cerebral cortex | Mouse | 8.35 | 5797 | 2698 | 10x Genomics (https://www.10xgenomics.com/resources/datasets) |
| Mouse brain sagittal anterior | Mouse | 7.34 | 6015 | 2696 | 10x Genomics (https://www.10xgenomics.com/resources/datasets) |
| Mouse brain sagittal posterior | Mouse | 6.97 | 4564 | 3339 | 10x Genomics (https://www.10xgenomics.com/resources/datasets) |
| Mouse kidney | Mouse | 7.70 | 5799 | 1434 | 10x Genomics (https://www.10xgenomics.com/resources/datasets) |
| HCC-1N | Human | 10.93 | 2651 | 2956 | GSA (https://ngdc.cncb.ac.cn/gsa-human/browse/HRA000437) |
| HCC-1L | Human | 15.16 | 4017 | 2791 | GSA (https://ngdc.cncb.ac.cn/gsa-human/browse/HRA000437) |
| HCC-1T | Human | 18.80 | 4790 | 3184 | GSA (https://ngdc.cncb.ac.cn/gsa-human/browse/HRA000437) |
| cHC-1N | Human | 14.08 | 2732 | 2207 | GSA (https://ngdc.cncb.ac.cn/gsa-human/browse/HRA000437) |
| cHC-1L | Human | 3.71 | 4193 | 4516 | GSA (https://ngdc.cncb.ac.cn/gsa-human/browse/HRA000437) |
| cHC-1T | Human | 1.67 | 5019 | 4779 | GSA (https://ngdc.cncb.ac.cn/gsa-human/browse/HRA000437) |
| HCC-2N | Human | 6.06 | 1405 | 4628 | GSA (https://ngdc.cncb.ac.cn/gsa-human/browse/HRA000437) |
| HCC-2L | Human | 3.90 | 2876 | 4672 | GSA (https://ngdc.cncb.ac.cn/gsa-human/browse/HRA000437) |
| HCC-2T | Human | 2.94 | 3366 | 4733 | GSA (https://ngdc.cncb.ac.cn/gsa-human/browse/HRA000437) |
| HCC-2P | Human | 2.67 | 3059 | 4666 | GSA (https://ngdc.cncb.ac.cn/gsa-human/browse/HRA000437) |
| HCC-3N | Human | 3.58 | 2905 | 4289 | GSA (https://ngdc.cncb.ac.cn/gsa-human/browse/HRA000437) |
| HCC-3L | Human | 2.13 | 3043.5 | 4758 | GSA (https://ngdc.cncb.ac.cn/gsa-human/browse/HRA000437) |
| HCC-3T | Human | 1.83 | 4058 | 4456 | GSA (https://ngdc.cncb.ac.cn/gsa-human/browse/HRA000437) |
| HCC-4N | Human | 6.16 | 622 | 4397 | GSA (https://ngdc.cncb.ac.cn/gsa-human/browse/HRA000437) |
| HCC-4L | Human | 6.81 | 3861 | 4113 | GSA (https://ngdc.cncb.ac.cn/gsa-human/browse/HRA000437) |
| HCC-4T | Human | 6.26 | 4276.5 | 4162 | GSA (https://ngdc.cncb.ac.cn/gsa-human/browse/HRA000437) |
| ICC-1L | Human | 2.79 | 4647.5 | 4654 | GSA (https://ngdc.cncb.ac.cn/gsa-human/browse/HRA000437) |
| HCC-5A | Human | 11.71 | 2024.5 | 3460 | GSA (https://ngdc.cncb.ac.cn/gsa-human/browse/HRA000437) |
| HCC-5B | Human | 11.60 | 1406 | 3958 | GSA (https://ngdc.cncb.ac.cn/gsa-human/browse/HRA000437) |
| HCC-5C | Human | 15.67 | 1501 | 3777 | GSA (https://ngdc.cncb.ac.cn/gsa-human/browse/HRA000437) |
| HCC-5D | Human | 4.08 | 1473.5 | 4352 | GSA (https://ngdc.cncb.ac.cn/gsa-human/browse/HRA000437) |

*Note*: Percentage of the UMI counts from the blank area in the whole section is calculated by the proportion of UMI counts in blank area to UMI counts in whole slice. UMI, unique molecular identifiers.
