# Supplementary material for: TIST: Transcriptome and Histopathological Image Integrative Analysis for Spatial Transcriptomics
Source: Genomics Proteomics Bioinformatics. 2022 Dec 19;20(5):974–88. doi: 10.1016/j.gpb.2022.11.012 (PMC10025771; doi:10.1016/j.gpb.2022.11.012)
Supplement: Supplementary Table S2 — Region names in the mouse cerebral cortex from the reference atlas diagram from Allen Institute for Brain Science [file mmc14.docx]

**Table S2 Region names in the mouse cerebral cortex from the reference atlas diagram from Allen Institute for Brain Science**

| **Region name abbreviated** | **Region name** |
| --- | --- |
| BS-IB-DORpm | Brain Stem-InterBrain-thalamus, polymodal association cortex related |
| BS-IB-DORpm-RT+GENd | Brain Stem-InterBrain-thalamus, polymodal association cortex related-Reticular nucleus of the Thalamus and geniculate group, ventral thalamus |
| BS-IB-DORsm | Brain Stem-InterBrain-thalamus, sensory-motor cortex related |
| BS-IB-HY | Brain Stem-InterBrain-HYpothalamus |
| CNU | Cerebral NUclei |
| CTX-HPF-HIP | Cerebral corTeX-Hippocampal formation-Hippocampal region |
| CTX-HPF-HIP-CA-sp | Cerebral corTeX-Hippocampal formation-Hippocampal region-Cornu Ammonis-pyramidal layer |
| CTX-HPF-HIP-DG-sg | Cerebral corTeX-Hippocampal formation-Hippocampal region- Dentate Gyrus- granule cell layer |
| CTX-Isocortex-inner | Cerebral corTeX-Isocortex-Inner layer |
| CTX-Isocortex-outer | Cerebral corTeX-Isocortex-Outer layer |
| CTX-OLF-PIR | Cerebral corTeX-OLFactory areas-PIRiform area |
| VS-VL | Ventricular Systems-lateral ventricle |
| fiber_tracts-alv+v3 | Fiber tracts-alveus and third ventricle |
| fiber_tracts-cpd | fiber tracts-cerebral peduncle |
| fiber_tracts-lfbs+mfbs | fiber tracts-lateral forebrain bundle system and medial forebrain bundle system |
| fiber_tracts-sm | fiber tracts-stria medullaris |
| CTX-Isocortex-middle | Cerebral corTeX-Isocortex-Middle layer |
