# Supplementary material for: TIST: Transcriptome and Histopathological Image Integrative Analysis for Spatial Transcriptomics
Source: Genomics Proteomics Bioinformatics. 2022 Dec 19;20(5):974–88. doi: 10.1016/j.gpb.2022.11.012 (PMC10025771; doi:10.1016/j.gpb.2022.11.012)
Supplement: Supplementary Table S3 — Comparison among TIST and state-of-the-art SC identification methods [file mmc15.docx]

**Table S3 Comparison among TIST and state-of-the-art SC identification methods**

| **Method** | **Spatial information integrated** | **Histology image used** | **Gene expression enhancement** | **Data denosing** | **Adaptively learn spatial similarity** |
| --- | --- | --- | --- | --- | --- |
| TIST | √ | √ | √ | √ | √ |
| stLearn | √ | √ |  | √ |  |
| SpaGCN | √ | √ |  |  |  |
| Louvain |  |  |  |  |  |
| BayesSpace | √ |  |  |  |  |
| STAGATE | √ |  |  | √ | √ |
| STEEL | √ |  |  |  |  |
| SEDR | √ |  |  |  |  |

*Note*: ST, spatial transcriptomics; SCs, spatial clusters; TIST, transcriptome and histopathological image integrative analysis for ST.
