# Supplementary material for: TIST: Transcriptome and Histopathological Image Integrative Analysis for Spatial Transcriptomics
Source: Genomics Proteomics Bioinformatics. 2022 Dec 19;20(5):974–88. doi: 10.1016/j.gpb.2022.11.012 (PMC10025771; doi:10.1016/j.gpb.2022.11.012)
Supplement: Supplementary Table S4 — Recovery results of the 49 genes used for evaluation in Figure 4C [file mmc16.docx]

**Table S4** **Recovery results of the 49 genes used for evaluation in Figure 4C**

| **Gene** | **Dropout rate** | | | | | | |
| --- | --- | --- | --- | --- | --- | --- | --- |
|  | **0.1** | **0.2** | **0.3** | **0.4** | **0.5** | **0.6** | **0.7** |
|  | **Recovery rate** | | | | | | |
| *1110008P14Rik* | 1 | 1 | 1 | 1 | 0.67 | 0.36 | 0.03 |
| *6330403K07Rik* | 1 | 1 | 1 | 0.98 | 0.51 | 0.28 | 0.01 |
| *Adarb1* | 1 | 1 | 1 | 1 | 1 | 0.73 | 0.29 |
| *Adcy1* | 1 | 1 | 1 | 1 | 0.74 | 0.38 | 0.08 |
| *Ahi1* | 1 | 1 | 1 | 1 | 0.93 | 0.57 | 0.2 |
| *Arpc5* | 1 | 1 | 1 | 1 | 1 | 1 | 0.26 |
| *Arpp21* | 0.82 | 0.71 | 0.62 | 0.43 | 0.26 | 0.1 | 0.02 |
| *Atp2b1* | 1 | 1 | 1 | 1 | 1 | 0.51 | 0.13 |
| *Baiap3* | 0.36 | 0.38 | 0.41 | 0.48 | 0.56 | 0.59 | 0.8 |
| *C1ql2* | 0.5 | 0.55 | 0.6 | 0.69 | 0.8 | 1 | 1 |
| *Camk2n1* | 1 | 1 | 1 | 0.5 | 0.09 | 0.05 | 0.55 |
| *Ccdc153* | 1 | 1 | 1 | 1 | 1 | 1 | 1 |
| *Cnp* | 1 | 1 | 1 | 1 | 1 | 0.77 | 0.35 |
| *Col1a2* | 0.29 | 0.3 | 0.36 | 0.46 | 0.52 | 0.62 | 0.95 |
| *Crlf1* | 0.43 | 0.45 | 0.49 | 0.52 | 0.66 | 0.78 | 0.87 |
| *Ctxn3* | 0.31 | 0.36 | 0.4 | 0.49 | 0.57 | 0.68 | 0.99 |
| *Dclk1* | 1 | 1 | 1 | 1 | 0.91 | 0.59 | 0.15 |
| *Ddn* | 0.49 | 0.44 | 0.35 | 0.26 | 0.13 | 0.04 | 0 |
| *Dsp* | 1 | 1 | 1 | 1 | 1 | 1 | 1 |
| *Dynlrb2* | 1 | 1 | 1 | 1 | 1 | 1 | 1 |
| *Enpp2* | 1 | 1 | 1 | 1 | 1 | 0.84 | 0.49 |
| *Epop* | 0.31 | 0.28 | 0.26 | 0.24 | 0.23 | 0.25 | 0.27 |
| *Fam163b* | 0.34 | 0.29 | 0.27 | 0.19 | 0.11 | 0.06 | 0.06 |
| *Folr1* | 0.91 | 0.96 | 1 | 1 | 1 | 1 | 1 |
| *Hap1* | 0.35 | 0.26 | 0.21 | 0.14 | 0.07 | 0.02 | 0 |
| *Hpca* | 1 | 1 | 0.97 | 0.67 | 0.41 | 0.14 | 0.06 |
| *Kcne2* | 1 | 1 | 1 | 1 | 1 | 1 | 1 |
| *Kl* | 0.34 | 0.36 | 0.39 | 0.44 | 0.5 | 0.6 | 0.67 |
| *Klk8* | 0.42 | 0.47 | 0.52 | 0.62 | 0.68 | 0.74 | 1 |
| *Lypd1* | 0.58 | 0.51 | 0.4 | 0.24 | 0.15 | 0.05 | 0.01 |
| *Mal* | 1 | 1 | 1 | 0.92 | 0.65 | 0.33 | 0.14 |
| *Mbp* | 1 | 1 | 0.9 | 0.44 | 0.19 | 0.13 | 0.47 |
| *Mef2c* | 0.79 | 0.65 | 0.57 | 0.46 | 0.33 | 0.16 | 0.04 |
| *Mgp* | 0.42 | 0.35 | 0.27 | 0.17 | 0.19 | 0.08 | 0.07 |
| *Mobp* | 1 | 1 | 0.94 | 0.74 | 0.52 | 0.34 | 0.19 |
| *Ndn* | 1 | 1 | 1 | 1 | 0.85 | 0.36 | 0.01 |
| *Nptxr* | 1 | 0.96 | 0.74 | 0.53 | 0.34 | 0.1 | 0.08 |
| *Plp1* | 1 | 1 | 1 | 0.8 | 0.41 | 0.24 | 0.27 |
| *Ptgds* | 1 | 1 | 1 | 1 | 0.52 | 0.24 | 0.33 |
| *Rasgrf2* | 0.3 | 0.24 | 0.2 | 0.13 | 0.08 | 0.04 | 0.05 |
| *Resp18* | 1 | 1 | 1 | 0.99 | 0.6 | 0.3 | 0.04 |
| *Rora* | 1 | 1 | 1 | 1 | 0.73 | 0.55 | 0.23 |
| *Slc6a11* | 1 | 0.94 | 0.82 | 0.7 | 0.43 | 0.2 | 0.07 |
| *Slc6a13* | 0.82 | 0.91 | 0.97 | 1 | 1 | 1 | 1 |
| *Snap25* | 1 | 1 | 0.81 | 0.39 | 0.06 | 0.08 | 0.51 |
| *Sparc* | 1 | 1 | 1 | 1 | 0.88 | 0.33 | 0.11 |
| *Spink8* | 0.38 | 0.45 | 0.5 | 0.55 | 0.57 | 0.75 | 1 |
| *Tmem212* | 1 | 1 | 1 | 1 | 1 | 1 | 1 |
| *Ttr* | 1 | 1 | 1 | 1 | 1 | 0.79 | 0.2 |
