# Supplementary material for: TIST: Transcriptome and Histopathological Image Integrative Analysis for Spatial Transcriptomics
Source: Genomics Proteomics Bioinformatics. 2022 Dec 19;20(5):974–88. doi: 10.1016/j.gpb.2022.11.012 (PMC10025771; doi:10.1016/j.gpb.2022.11.012)
Supplement: Supplementary Table S5 — Previously published evidence of significant ligand-receptor pairs marked in Figure 6B [file mmc17.docx]

| **Ligand gene** | **Receptor gene** | **Identified SC** | **Literature report** | **Pubmed ID** |
| --- | --- | --- | --- | --- |
| *Wnt2* | *Fzd3+Lrp6* | SC_3, SC_9 | Activates intracellular signalling and facilitate brain development as well as adult hippocampal neurogenesis | 33042988 |
| *Wnt2* | *Fzd1+Lrp6* | SC_9 | Activates intracellular signalling and facilitate brain development as well as adult hippocampal neurogenesis | 33042988 |
| *Wnt10a* | *Fzd3+Lrp6* | SC_3, SC_9 | Activates intracellular signalling and facilitate brain development as well as adult hippocampal neurogenesis | 33042988 |
| *Wnt10a* | *Fzd1+Lrp6* | SC_9 | Activates intracellular signalling and facilitate brain development as well as adult hippocampal neurogenesis | 33042988 |
| *Tgfb2* | *Tgfbr1 + Tgfbr2* | SC_9 | Plays a critical role in late-stage adult neurogenesis | 24859199,  32433957 |
| *Tgfb2* | *Acvr1c + Tgfbr2* | SC_3, SC_9 | Participates in the rodent central nervous system during postnatal development. | 32433957,  8875430,  15485907 |
| *Tgfb2* | *Acvr1b + Tgfbr2* | SC_3, SC_9 | Participates in the rodent central nervous system during postnatal development. | 32433957,  8875430,  15485907 |
| *Tgfb2* | *Acvr1 + Tgfbr1* | SC_3, SC_9 | Participates in the rodent central nervous system during postnatal development. | 32433957,  8875430,  15485907 |
| *Ptn* | *Ptprz1* | SC_2 | Affects neurons by regulating dendritic length and complexity and spine density. | 30605653 |
| *Psap* | *Gpr37l1* | SC_2 | Participates in modulating the activity and function of neurons by acting with neuron-derived neurotrophic factor, potentially regulating memory encoding and retrieval. | 28795439 |
| *Npy* | *Npylr* | SC_3, SC_9 | Reduces glutamatergic neurotransmission onto pyramidal cells and involve in synaptic transmission in hippocampus. | 9334392,  12482942 |
| *Fgf5* | *Fgfr2* | SC_9 | Has biological roles in ranging from brain development, neuronal replacement to adult plasticity. | 8462006 |
| *Fgf5* | *Fgfr1* | SC_9 | Has biological roles in ranging from brain development, neuronal replacement to adult plasticity. | 8462006 |

**Table S5 Previously published evidence of significant ligand–receptor pairs marked in Figure 6B**
